# Ferric Chloride Promoted Glycosidation of Alkyl Thioglycosides

**DOI:** 10.3390/molecules29204845

**Published:** 2024-10-13

**Authors:** Lacie M. Ridgway, Anupama Das, Melanie L. Shadrick, Alexei V. Demchenko

**Affiliations:** Department of Chemistry, Saint Louis University, 3501 Laclede Ave., St. Louis, MO 63103, USA

**Keywords:** glycosylation, ferric chloride, green chemistry, carbohydrates, thioglycosides

## Abstract

Reported herein is a new reaction for glycosylation with thioglycosides in the presence of iron(III) chloride. Previously, FeCl_3_ was used for the activation of thioglycosides as a Lewis acid co-promoter paired with NIS. In the reported process, although 5.0 equiv of FeCl_3_ are needed to activate thioglycosides most efficiently, no additives were used, and the reactions with reactive glycosyl donors smoothly proceeded to completion in 1 h at 0 °C. This work showcases a new direction in developing glycosylation methods using greener and earth-abundant activators.

## 1. Introduction

Carbohydrates, the most abundant class of biomolecules, play a significant role in many fundamental processes such as energy storage, disease progression, and immune responses to viral and bacterial invasions [1]. Hence, access to these molecules is important for vaccine and pharmaceutical research and development. While synthetic strategies for glycosylation have been thoroughly investigated, the need for greener and less toxic methods is imperative to the advancement of glycochemistry. Among the glycosyl donors developed for chemical glycosylation, thioglycosides are a popular choice due to their stability and ability to withstand harsh conditions associated with protecting group manipulations. The activation of thioglycosides for glycosylation can be achieved by the use of electrophilic or thiophilic promotors, often under mild reaction conditions [2]. Among these, organosulfur compounds [3,4,5,6,7,8]; photo-activators [9,10,11,12]; and halogens [13,14,15,16,17,18] are the most popular. Activation with metal salts has also shown to be a promising direction in methodology development [19].

Metal salt activation was introduced by Ferrier [20] who employed mercury(II) salts. More recently, Pohl et al. demonstrated that stoichiometric amounts of Ph_3_Bi(OTf)_2_ were effective in activating thioglycosides for glycosylation [21,22]. Further studies of metal salt-promoted glycosylations reported by Sureshan et al. employed donor activation using AuCl_3_ in sub-stoichiometric amounts [23]. Activation using Au(III) salts was also achieved by Zhu et al. through direct coordination of Au(III) on the sulfur atom [24]. These methodological studies indicated that transition metal activation of thioglycosides can lead to promising regio- and stereoselectivity and provide good yields. To further the advancement of transition metals as promotors in thioglycosides, our group previously reported the use of palladium(II) bromide and copper(II) bromide to activate aryl/alkylthio glycosides [25,26]. While successful, the need to identify greener, cheaper, and more accessible transition metal salts capable of activating thioglycosides remains vital to the expansion of glycochemistry.

Iron(III) chloride, a salt formed with the second most abundant metal on earth, is naturally abundant, inexpensive, and relatively benign [27]. Ferric chloride has been applied in the introduction of protecting groups in carbohydrates [28,29]. The application of FeCl_3_ in *O*-glycosylation has also emerged, most commonly for the activation of glycosyl donors bearing the anomeric acetate group [30,31,32,33,34,35,36,37,38,39]. Other applications for the activation of aryl glycoside [40], pivaloate [41], bromide [42], imidate [43], chloride [44], or propargyl glycoside [45] have also been explored. FeCl_3_ has also been employed as a co-catalyst for the activation of hemiacetal donors [46] and aryl-thioglycosides in the presence of *N*-iodosuccinimide [43,47]. Herein, we report a new protocol for efficient and versatile glycosidation of thioglycosides in the presence of iron(III) chloride without any co-promoters or additives.

## 2. Results and Discussion

For the preliminary study, we selected per-*O*-benzylated (armed) ethylthio galactosyl donor **1** which was reacted with primary glycosyl acceptor **2** in the presence of molecular sieves (3 Å) at 0 °C. Glycosylation in the presence of catalytic amount of FeCl_3_ (0.2 equiv) in DCM was ineffective (entry 1, Table 1). However, when the amount of FeCl_3_ was increased to 1.0 equiv, disaccharide **3** was obtained, albeit in low yield: 12% for the reaction in DCM and 15% for the reaction in MeCN (entries 2 and 3). Interestingly, when the reaction was carried out in a DCM/MeCN (1/1, *v*/*v*) mixture, the yield was doubled in respect to reactions wherein either solvent was used individually (24%, entry 4).

When the amount of FeCl_3_ was further increased to 2.0 equiv, disaccharide **3** was obtained in improved yields, which were practically identical for both individual solvents: 23% for the reaction in DCM and 22% for the reaction in MeCN (entries 5 and 6). Again, when the reaction was carried out in DCM/MeCN (1/1, *v*/*v*) solvent mixture, the yield for the formation of disaccharide **3** is doubled (54%, entry 7). The reaction was stopped after 1 h, and prolonged experiments conducted over 16 or 24 h provided identical yields. Investigations into the effects of temperature did not offer any gains at −30 °C, rt, or 40 °C, and was detrimental to yields. Regardless of the reaction solvent, some anomerization of donor **1** into its α-linked counterpart was observed in the majority of experiments.

With DCM/MeCN as the reaction solvent at 0 °C, we performed glycosylation in the presence of 3.0 equiv FeCl_3_ to afford disaccharide **3** in an improved yield of 64% in 1 h (entry 8). When the amount of FeCl_3_ was increased to 5.0 equiv, the reaction smoothly proceeded to completion in 1 h, and disaccharide **3** was isolated in a high yield of 96% (entry 9). We note that although the reaction was performed in the presence of MeCN, which is known to be a β-directing solvent, the β-anomer was only slightly favored in this reaction (α/β = 1/1.5).

With the most favorable reaction conditions established, FeCl_3_ (5 equiv), DCM/MeCN (1/1, *v*/*v*), and molecular sieves (3 Å) at 0 °C (entry 9, Table 1), we proceeded with investigating other glycosylation reactions using different donor–acceptor combinations. The outcome of this study is summarized in Table 2. The activation of glycosyl donor **1** for reactions with secondary glycosyl acceptors **4**, **6**, and **8** produced respective disaccharides **5**, **7**, and **9** in good to excellent yields of 73–94% in 1 h (entries 1–3). The highest yield of 94% was obtained for glycosylation of 3-OH glycosyl acceptor **6** (entry 2). We note that although MeCN is a known β-directing solvent, we observed no stereoselectivity in these reactions (α/β from 1.6/1 to 1/1.8).

Glycosidation of galactosyl donor **10** equipped with the super-arming protecting group pattern, 2-*O*-benzoyl-3,4,6-tri-*O*-benzyl, also worked well in reactions with glycosyl acceptors **2**, **4**, **6**, and **8**, and the respective disaccharides **11**–**14** were obtained in good to excellent yields of 68–94% (entries 4–7). The highest identical yields of 94% were obtained for glycosylations of 6-OH and 3-OH glycosyl acceptors **2** and **6** (entries 4 and 6). These glycosylations were all β-selective due to the neighboring 2-*O*-benzoyl group participation.

Along similar lines, we investigated per-*O*-benzylated glucosyl donor **15**. These glycosylations also worked very efficiently with glycosyl acceptors **2**, **4**, **6**, and **8,** and the respective disaccharides **16**–**19** were obtained in excellent yields of 80–92% (entries 8–11). We noticed some preference for β-selectivity in this series (α/β = 1/2.6–4.1), probably due to the effect of MeCN, a known participating solvent.

Glycosidation of glucosyl donor **20** equipped with the super-arming protecting group pattern, 2-*O*-benzoyl-3,4,6-tri-*O*-benzyl, also worked well in reactions with glycosyl acceptors **2**, **4**, **6**, and **8**. The respective disaccharides **21**–**24** were obtained in good to excellent yields of 71–91% (entries 12–15). The highest yields of 87–91% were obtained for glycosylations of 6-OH and 2-OH glycosyl acceptors **2** and **4** (entries 12 and 13). These glycosylations were all β-selective due to the neighboring group participation of 2-*O*-benzoyl substituent. Finally, we investigated the less reactive per-*O*-benzoylated galactosyl donor **25**. Disaccharide **26** was obtained in a good yield of 77% with exclusive β-selectivity (entry 16).

## 3. Materials and Methods

### 3.1. General Methods

The reactions were performed using commercial reagents and the ACS grade solvents used for reactions were purified and dried in accordance with standard procedures. Column chromatography was performed on silica gel 60 (70–230 mesh); reactions were monitored by TLC on Kieselgel 60 F_254_. The compounds were detected by examination under UV light and by charring with 10% sulfuric acid in methanol. Solvents were removed under reduced pressure at <40 °C. CH_2_Cl_2_ was distilled from CaH_2_ directly prior to application. Molecular sieves (3 Å) used for reactions were crushed and activated in vacuo at 390 °C for 8 h in the first instance and then for 2–3 h at 390 °C directly prior to application. ^1^H NMR spectra were recorded at 400 MHz (Bruker); ^13^C NMR spectra were recorded at 100 MHz. The ^1^H NMR chemical shifts are referenced to tetramethyl silane (TMS, δ = 0 ppm) or CDCl_3_ (CHCl_3_ δ = 7.26 ppm) for ^1^H NMR spectra for solutions in CDCl_3_. The ^13^C NMR chemical shifts are referenced to the central signal of CDCl_3_ (δ = 77.00 ppm) for solutions in CDCl_3_. To assist structural assignments, further information was obtained utilizing gCOSY and gHSQC experiments. Anomeric ratios (if applicable) were determined by comparison of the integral intensities of relevant signals in ^1^H NMR spectra (see the Supporting Information).

### 3.2. Synthesis of Building Blocks

**Ethyl 2,3,4,6-tetra-*O*-benzyl-1-thio-β-D-galactopyranoside (1)** was synthesized as reported previously and its analytical data were in accordance with that previously described [15,48].

**Methyl 2,3,4-tri-*O*-benzyl-α-D-glucopyranoside (2)** was synthesized as reported previously and its analytical data were in accordance with that previously described [49].

**Methyl 3,4,6-tri-*O*-benzyl-α-D-glucopyranoside (4)** was synthesized as reported previously and its analytical data were in accordance with that previously described [49].

**Methyl 2,4,6-tri-*O*-benzyl-α-D-glucopyranoside (6)** was synthesized as reported previously and its analytical data were in accordance with that previously described [49,50].

**Methyl 2,3,6-tri-*O*-benzyl-α-D-glucopyranoside (8)** was synthesized as reported previously and its analytical data were in accordance with that previously described [49].

**Ethyl 2-*O*-benzoyl-3,4,6-tri-*O*-benzyl-1-thio-β-D-galactopyranoside (10)** was synthesized as reported previously and its analytical data were in accordance with that previously described [51,52].

**Ethyl 2,3,4,6-tetra-*O*-benzyl-1-thio-β-D-glucopyranoside (15)** was synthesized as reported previously and its analytical data were in accordance with that previously described [53].

**Ethyl 2-*O*-benzoyl-3,4,6-tri-*O*-benzyl-1-thio-β-D-glucopyranoside (20)** was synthesized as reported previously and its analytical data were in accordance with that previously described [52,54].

**Ethyl 2,3,4,6-tetra-*O*-benzoyl-1-thio-β-D-galactopyranoside (25)** was synthesized as reported previously and its analytical data were in accordance with that previously described [55].

### 3.3. Synthesis of Disaccharides

**General procedure for glycosidation.** A mixture containing thioglycoside donor (50 mg, 0.078–0.086 mmol), glycosyl acceptor (0.062–0.071 mmol), and freshly activated molecular sieves (3 Å, 300 mg) in CH_2_Cl_2_/CH_3_CN (1.0 mL, 1/1, *v*/*v*) was stirred under argon for 2 h at rt. The reaction mixture was cooled to 0 °C, anhydrous ferric chloride (FeCl_3_, 0.390–0.430 mmol, 5.0 equiv to donor) was added, and the resulting mixture was stirred under argon for 1 h at 0 °C. After that, sat. aq. NaHCO_3_ (3 mL) was added and the resulting mixture was stirred for 10 min. The solids were filtered off through a pad of Celite and rinsed successively with CH_2_Cl_2_. The combined filtrate (~15 mL) was concentrated under reduced pressure. The residue was diluted with DCM (5 mL) and washed with H_2_O (5 mL) and brine (2 × 5 mL). The organic phase was separated, dried with Na_2_SO_4_, and concentrated under reduced pressure. The residue was purified by column chromatography on silica gel (ethyl acetate-hexane gradient elution or acetone-toluene gradient elution) to afford a disaccharide derivative in yields listed in tables and below.

**Methyl 2,3,4-tri-*O*-benzyl-6-*O*-(2,3,4,6-tetra-*O*-benzyl-α/β-D-galactopyranosyl)-α-D-glucopyranoside (3)** was obtained from thioglycoside **1** and glycosyl acceptor **2** by the general glycosylation method in 96% yield (α/β = 1/1.5) as a colorless syrup. Analytical data for **3** were in accordance with that reported previously [56].

**Methyl 2-*O*-(2,3,4,6-tetra-*O*-benzyl-α/β-D-galactopyranosyl)-3,4,6-tri-*O*-benzyl-α-D-glucopyranoside (5)** was obtained from thioglycoside **1** and glycosyl acceptor **4** by the general glycosylation method in 89% yield (α/β = 1.6/1) as a colorless syrup. Analytical data for **5** were in accordance with that reported previously [57].

**Methyl 2,4,6-tri-*O*-benzyl-3-*O*-(2,3,4,6-tetra-*O*-benzyl-α/β-D-galactopyranosyl)-α-D-glucopyranoside (7)** was obtained from thioglycoside **1** and glycosyl acceptor **6** by the general glycosylation method in 94% yield (α/β = 1/1.4) as a colorless syrup. Analytical data for **7** were in accordance with that reported previously [58].

**Methyl 2,3,6-tri-*O*-benzyl-4-*O*-(2,3,4,6-tetra-*O*-benzyl-α/β-D-galactopyranosyl)-α-D-glucopyranoside (9)** was obtained from thioglycoside **1** and glycosyl acceptor **8** by the general glycosylation method in 73% yield (α/β = 1/1.8) as a colorless syrup. Analytical data for **9** were in accordance with that reported previously [59].

**Methyl 6-*O*-(2-*O*-benzoyl-3,4,6-tri-*O*-benzyl-β-D-galactopyranosyl)-2,3,4-tri-*O*-benzyl-α-D-glucopyranoside (11)** was obtained from thioglycoside **10** and glycosyl acceptor **2** by the general glycosylation method in 94% yield (β only) as a white amorphous solid. Analytical data for **11** were in accordance with that reported previously [60].

**Methyl 2-*O*-(2-*O*-benzoyl-3,4,6-tri-*O*-benzyl-β-D-galactopyranosyl)-3,4,6-tri-*O*-benzyl-α-D-glucopyranoside (12)** was obtained from thioglycoside **10** and glycosyl acceptor **4** by the general glycosylation method in 77% yield (β only) as a white amorphous solid. Analytical data for **12** were in accordance with that reported previously [25].

**Methyl 3-*O*-(2-*O*-benzoyl-3,4,6-tri-*O*-benzyl-β-D-galactopyranosyl)-2,4,6-tri-*O*-benzyl-α-D-glucopyranoside (13)** was obtained from thioglycoside **10** and glycosyl acceptor **6** by the general glycosylation method in 94% yield (β only) as a white amorphous solid. Analytical data for 1**3** were in accordance with that reported previously [60].

**Methyl 4-*O*-(2-*O*-benzoyl-3,4,6-tri-*O*-benzyl-β-D-galactopyranosyl)-2,3,6-tri-*O*-benzyl-α-D-glucopyranoside (14)** was obtained from thioglycoside **10** and glycosyl acceptor **8** by the general glycosylation method in 68% yield (β only) as a white amorphous solid. Analytical data for **14** were in accordance with that reported previously [60].

**Methyl 2,3,4-tri-*O*-benzyl-6-*O*-(2,3,4,6-tetra-*O*-benzyl-α/β-D-glucopyranosyl)-α-D-glucopyranoside (16)** was obtained from thioglycoside **15** and glycosyl acceptor **2** by the general glycosylation method in 92% yield (α/β = 1/4.1) as a white amorphous solid. Analytical data for **16** were in accordance with that reported previously [61].

**Methyl 2-*O*-(2,3,4,6-tetra-*O*-benzyl-α/β-D-glucopyranosyl)-3,4,6-tri-*O*-benzyl-α-D-glucopyranoside (17)** was obtained from thioglycoside **15** and glycosyl acceptor **4** by the general glycosylation method in 89% yield, in which α was obtained in 17.81 mg and β in 44.68 mg (α/β = 1/2.6) as a white amorphous solid. Analytical data for **17** were in accordance with that reported previously [62].

**Methyl 2,4,6-tri-*O*-benzyl-3-*O*-(2,3,4,6-tetra-*O*-benzyl-α/β-D-glucopyranosyl)-α-D-glucopyranoside (18)** was obtained from thioglycoside **15** and glycosyl acceptor **6** by the general glycosylation method in 80% yield (α/β = 1/2.8) as a white amorphous solid. Analytical data for **18** were in accordance with that reported previously [63].

**Methyl 2,3,6-tri-*O*-benzyl-4-*O*-(2,3,4,6-tetra-*O*-benzyl-α/β-D-glucopyranosyl)-α-D-glucopyranoside (19)** was obtained from thioglycoside **15** and glycosyl acceptor **8** by the general glycosylation method in 85% yield (α/β = 1/3.6) as a white amorphous solid. Analytical data for **19** were in accordance with that reported previously [64].

**Methyl 6-*O*-(2-*O*-benzoyl-3,4,6-tri-*O*-benzyl-β-D-glucopyranosyl)-2,3,4-tri-*O*-benzyl-α-D-glucopyranoside (21)** was obtained from thioglycoside **20** and glycosyl acceptor **2** by the general glycosylation method in 87% yield (β only) as a white amorphous solid. Analytical data for **21** were in accordance with that reported previously [65].

**Methyl 2-*O*-(2-*O*-benzoyl-3,4,6-tri-*O*-benzyl-β-D-glucopyranosyl)-3,4,6-tri-*O*-benzyl-α-D-glucopyranoside (22)** was obtained from thioglycoside **20** and glycosyl acceptor **4** by the general glycosylation method in 91% yield (β only) as a white amorphous solid. Analytical data for **22** were in accordance with that reported previously [66].

**Methyl 3-*O*-(2-*O*-benzoyl-3,4,6-tri-*O*-benzyl-β-D-glucopyranosyl)-2,4,6-tri-*O*-benzyl-α-D-glucopyranoside (23)** was obtained from thioglycoside 20 and glycosyl acceptor **6** by the general glycosylation method in 80% yield (β only) as a white amorphous solid. Analytical data for **23** were in accordance with that reported previously [66].

**Methyl 4-*O*-(2-*O*-benzoyl-3,4,6-tri-*O*-benzyl-β-D-glucopyranosyl)-2,3,6-tri-*O*-benzyl-α-D-glucopyranoside (24)** was obtained from thioglycoside **20** and glycosyl acceptor **8** by the general glycosylation method in 71% yield (β only) as a white amorphous solid. Analytical data for **24** were in accordance with that reported previously [66].

**Methyl 6-*O*-(2,3,4,6-tetra-*O*-benzoyl-β-D-galactopyranosyl)-2,3,4-tri-*O*-benzyl-α-D-glucopyranoside (26)** was obtained from thioglycoside **25** and glycosyl acceptor **2** by the general glycosylation method in 77% yield (β only) as a colorless syrup. Analytical data for **26** were in accordance with that reported previously [67].

## 4. Conclusions

A new method for the activation of alkyl thioglycosides using inexpensive and abundant FeCl_3_ has been developed. Upon optimization of the reaction conditions using the per-*O*-benzylated galactosyl donor, it was determined that efficient activation could be achieved using stoichiometric amounts of FeCl_3_ in a mixture of DCM/MeCN. Extension of these reaction conditions to armed and superarmed galactosyl and glucosyl donors with a series of primary and secondary glycosyl acceptors produced disaccharides in good to excellent yields. Broadening the scope of this method to glycosidation of less reactive glycosyl donors, investigation of the reaction mechanism, and application to the synthesis of glycans are currently underway in our laboratory.

## Figures and Tables

**Table 1:** FeCl_3_ promoted glycosidation of thioglycoside donor **1** with glycosyl acceptor **2**.

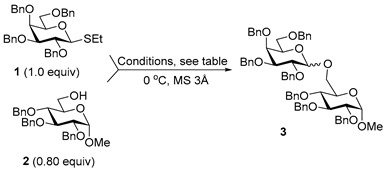		
**Entry**	**Conditions**	**Yield**
1	FeCl_3_ (0.2 equiv), DCM, 2 h	NR ^a^
2	FeCl_3_ (1.0 equiv), DCM, 2 h	**3**, ^b^ 12%
3	FeCl_3_ (1.0 equiv), MeCN, 2 h	**3**, 15%
4	FeCl_3_ (1.0 equiv), DCM/MeCN (1/1, *v*/*v*), 1 h	**3**, 24%
5	FeCl_3_ (2.0 equiv), DCM, 2 h	**3**, 23%
6	FeCl_3_ (2.0 equiv), MeCN, 2 h	**3**, 22%
7	FeCl_3_ (2.0 equiv), DCM/MeCN (1/1, *v*/*v*), 1 h	**3**, 54%
8	FeCl_3_ (3.0 equiv), DCM/MeCN (1/1, *v*/*v*), 1 h	**3**, 64%
9	FeCl_3_ (5.0 equiv), DCM/MeCN (1/1, *v*/*v*), 1 h	**3**, 96%

^a^ NR—no reaction; ^b^ in all reactions, compound **3** was obtained as a mixture of anomers.

**Table 2:** Expanding the scope of FeCl_3_ promoted glycosylation to other donors and acceptors ^a^.

Entry	Donor	Acceptor	Product, Yield, Ratio α/β
1	**1**	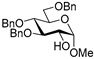 **4**	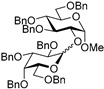 **5**, 89%, α/β = 1.6/1
2	**1**	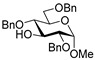 **6**	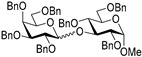 **7**, 94%, α/β = 1/1.4
3	**1**	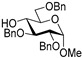 **8**	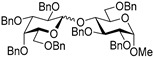 **9**, 73%, α/β = 1/1.8
4	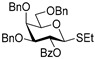 **10**	**2**	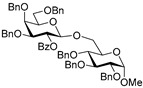 **11**, 94%, β only
5	**10**	**4**	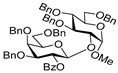 **12**, 77% β only
6	**10**	**6**	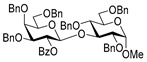 **13**, 94%, β only
7	**10**	**8**	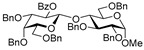 **14**, 68%, β only
8	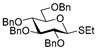 **15**	**2**	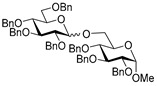 **16**, 92%, α/β = 1/4.1
9	**15**	**4**	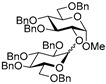 **17**, 89%, α/β = 1/2.6
10	**15**	**6**	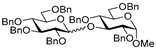 **18**, 80%, α/β = 1/2.8
11	**15**	**8**	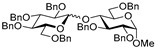 **19**, 85%, α/β = 1/3.6
12	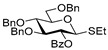 **20**	**2**	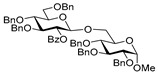 **21**, 87%, β only
13	**20**	**4**	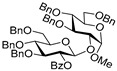 **22**, 91%, β only
14	**20**	**6**	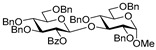 **23**, 80%, β only
15	**20**	**8**	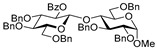 **24**, 71%, β only
16	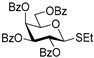 **25**	**2**	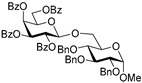 **26**, 77%, β only

^a^ Reactions were performed using benchmark conditions stated in Table 1 (entry 9).

## Data Availability

All data are available from the authors.

## Supplementary Materials

The following supporting information can be downloaded at: https://www.mdpi.com/article/10.3390/molecules29204845/s1, Figure S1. ^1^H NMR Spectrum (CDCl_3_, 400 MHz) of Compound 3; Figure S2. HSQC NMR Spectrum (CDCl_3_, 400 MHz) of Compound 3; Figure S3; ^1^H NMR Spectrum (CDCl_3_, 400 MHz) of Compound 5; Figure S4. HSQC NMR Spectrum (CDCl_3_, 400 MHz) of Compound 5; Figure S5. ^1^H NMR Spectrum (CDCl_3_, 400 MHz) of Compound 7; Figure S6. HSQC NMR Spectrum (CDCl_3_, 400 MHz) of Compound 7; Figure S7. ^1^H NMR Spectrum (CDCl_3_, 400 MHz) of Compound 9; Figure S8. HSQC NMR Spectrum (CDCl_3_, 400 MHz) of Compound 9; Figure S9. ^1^H NMR Spectrum (CDCl_3_, 400 MHz) of Compound 11; Figure S10. HSQC NMR Spectrum (CDCl_3_, 400 MHz) of Compound 11; Figure S11. ^1^H NMR Spectrum (CDCl_3_, 400 MHz) of Compound 12; Figure S12. HSQC NMR Spectrum (CDCl_3_, 400 MHz) of Compound 12; Figure S13. ^1^H NMR Spectrum (CDCl_3_, 400 MHz) of Compound 13; Figure S14. HSQC NMR Spectrum (CDCl_3_, 400 MHz) of Compound 13; Figure S15. ^1^H NMR Spectrum of (CDCl_3_, 400 MHz) Compound 14; Figure S16. HSQC NMR Spectrum (CDCl_3_, 400 MHz) of Compound 14; Figure S17. ^1^H NMR Spectrum (CDCl_3_, 400 MHz) of Compound 16; Figure S18. HSQC NMR Spectrum (CDCl_3_, 400 MHz) of Compound 16; Figure S19. ^1^H NMR Spectrum (CDCl_3_, 400 MHz) of Compound 17β; Figure S20. HSQC NMR Spectrum (CDCl_3_, 400 MHz) of Compound 17β; Figure S21. ^1^H NMR Spectrum (CDCl_3_, 400 MHz) of Compound 17α; Figure S22. HSQC NMR Spectrum (CDCl_3_, 400 MHz) of Compound 17α; Figure S23. ^1^H NMR Spectrum (CDCl_3_, 400 MHz) of Compound 18; Figure S24. HSQC NMR Spectrum (CDCl_3_, 400 MHz) of Compound 18; Figure S25. ^1^H NMR Spectrum (CDCl_3_, 400 MHz) of Compound 19; Figure S26. HSQC NMR Spectrum (CDCl_3_, 400 MHz) of Compound 19; Figure S27. ^1^H NMR Spectrum (CDCl_3_, 400 MHz) of Compound 21; Figure S28. HSQC NMR Spectrum (CDCl_3_, 400 MHz) of Compound 21; Figure S29. ^1^H NMR Spectrum (CDCl_3_, 400 MHz) of Compound 22; Figure S30. HSQC NMR Spectrum (CDCl_3_, 400 MHz) of Compound 22; Figure S31. ^1^H NMR Spectrum (CDCl_3_, 400 MHz) of Compound 23; Figure S32. HSQC NMR Spectrum (CDCl_3_, 400 MHz) of Compound 23; Figure S33. ^1^H NMR Spectrum (CDCl_3_, 400 MHz) of Compound 24; Figure S34. HSQC NMR Spectrum (CDCl_3_, 400 MHz) of Compound 24; Figure S35. ^1^H NMR Spectrum (CDCl_3_, 400 MHz) of Compound 26; Figure S36. HSQC NMR Spectrum (CDCl_3_, 400 MHz) of Compound 26.
