# Supplementary material for: Ferric Chloride Promoted Glycosidation of Alkyl Thioglycosides
Source: Molecules. 2024 Oct 13;29(20):4845. doi: 10.3390/molecules29204845 (PMC11510396; doi:10.3390/molecules29204845)
Supplement: Supplementary file 1 [file molecules-29-04845-s001.zip › molecules-3205341-supplementary.pdf]

# Ferric Chloride Promoted Glycosylation of Alkyl Thioglycosides

**Lacie M. Ridgway, Anupama Das, Melanie L. Shadrick, and Alexei V. Demchenko***

Department of Chemistry, Saint Louis University, 3501 Laclede Ave, St. Louis, MO 63103, USA; e-mail: alexei.demchenko@slu.edu

**NMR spectra of known compounds**

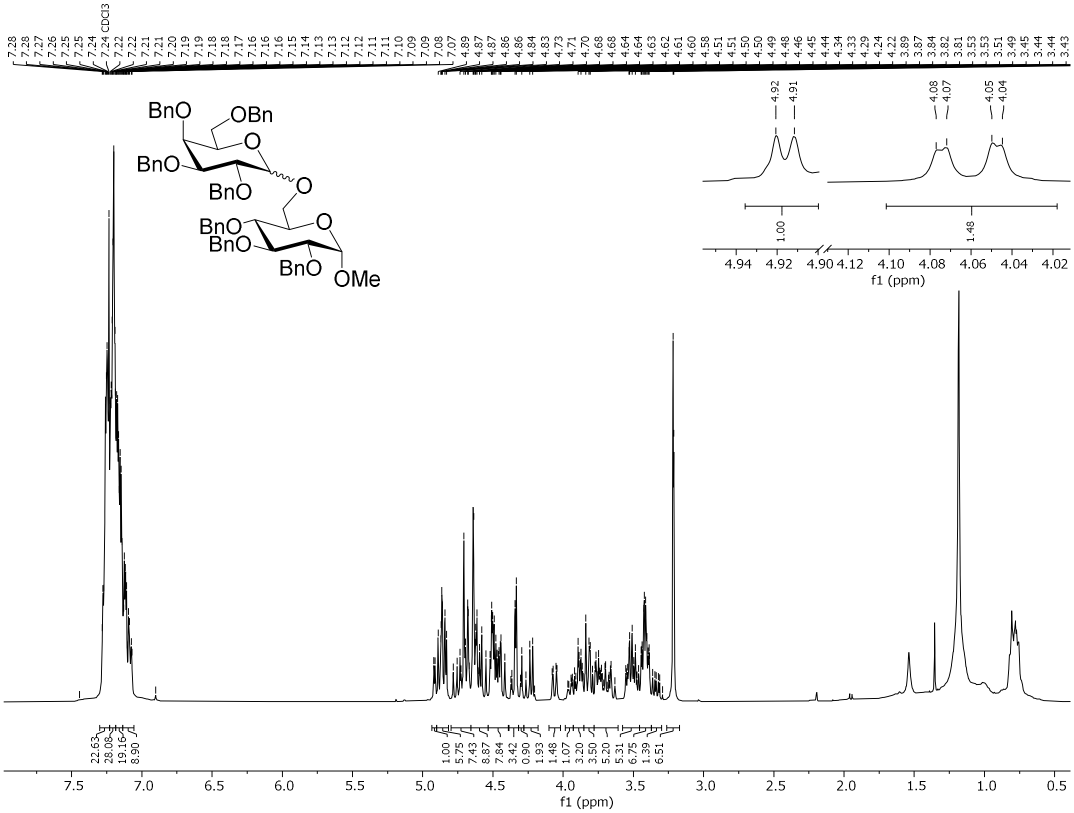


**Figure S1.** $^1$H NMR Spectrum ($CDCl_3$, 400 MHz) of Compound **3.**

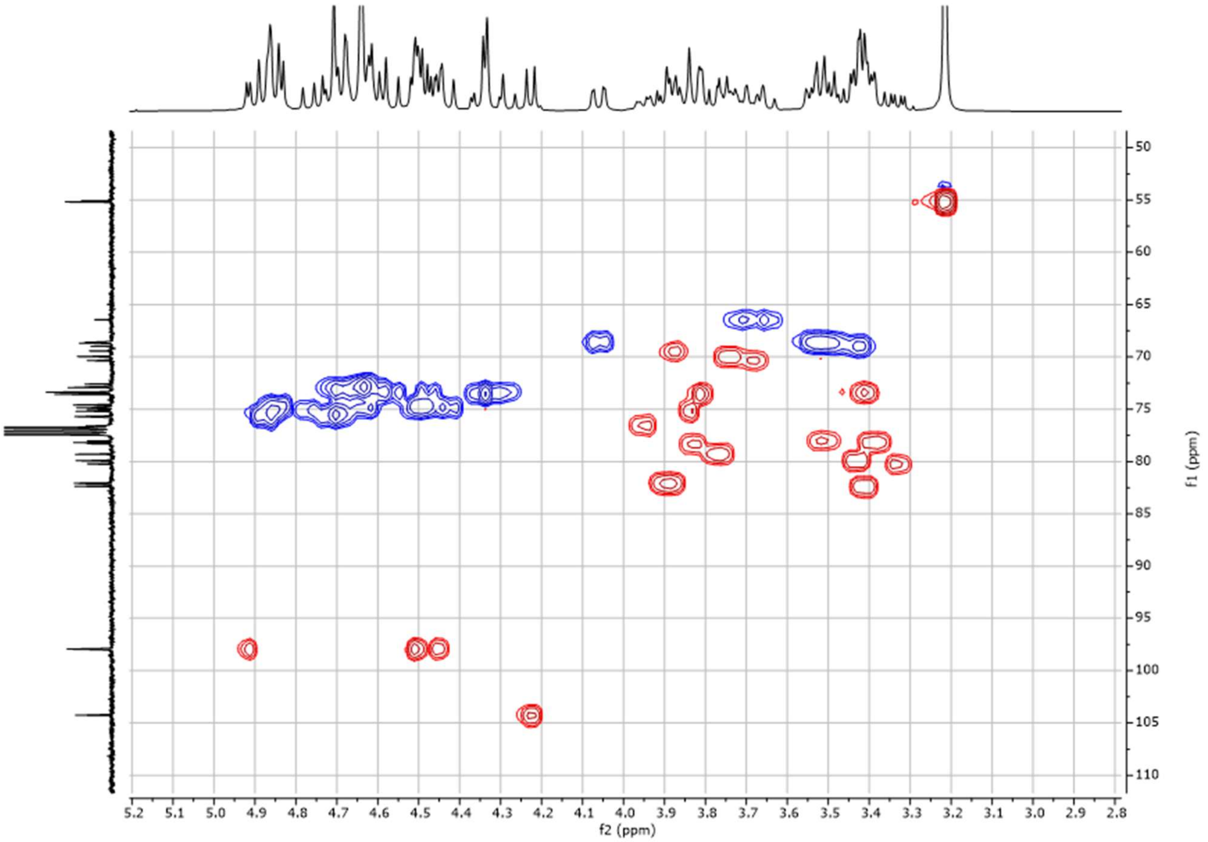


**Figure S2.** HSQC NMR Spectrum ($CDCl_3$, 400 MHz) of Compound **3**.

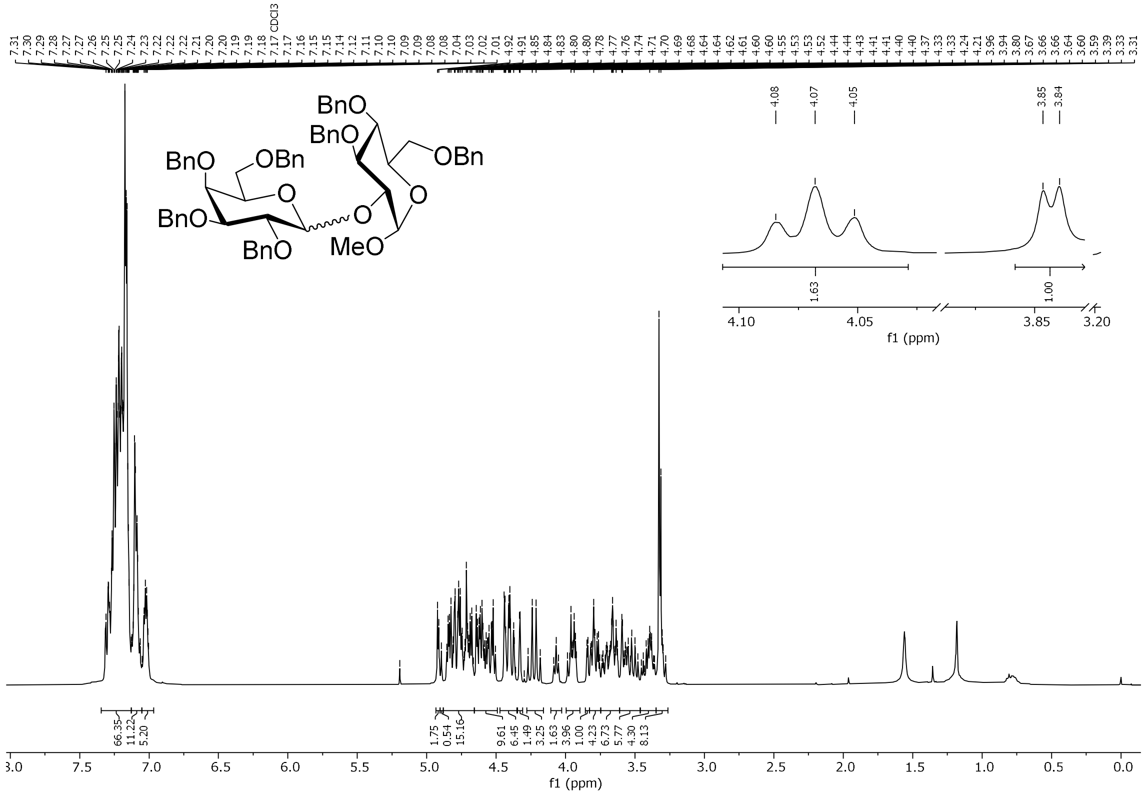


**Figure S3.** $^{1}H$ NMR Spectrum ($CDCl_3$, 400 MHz) of Compound **5**.

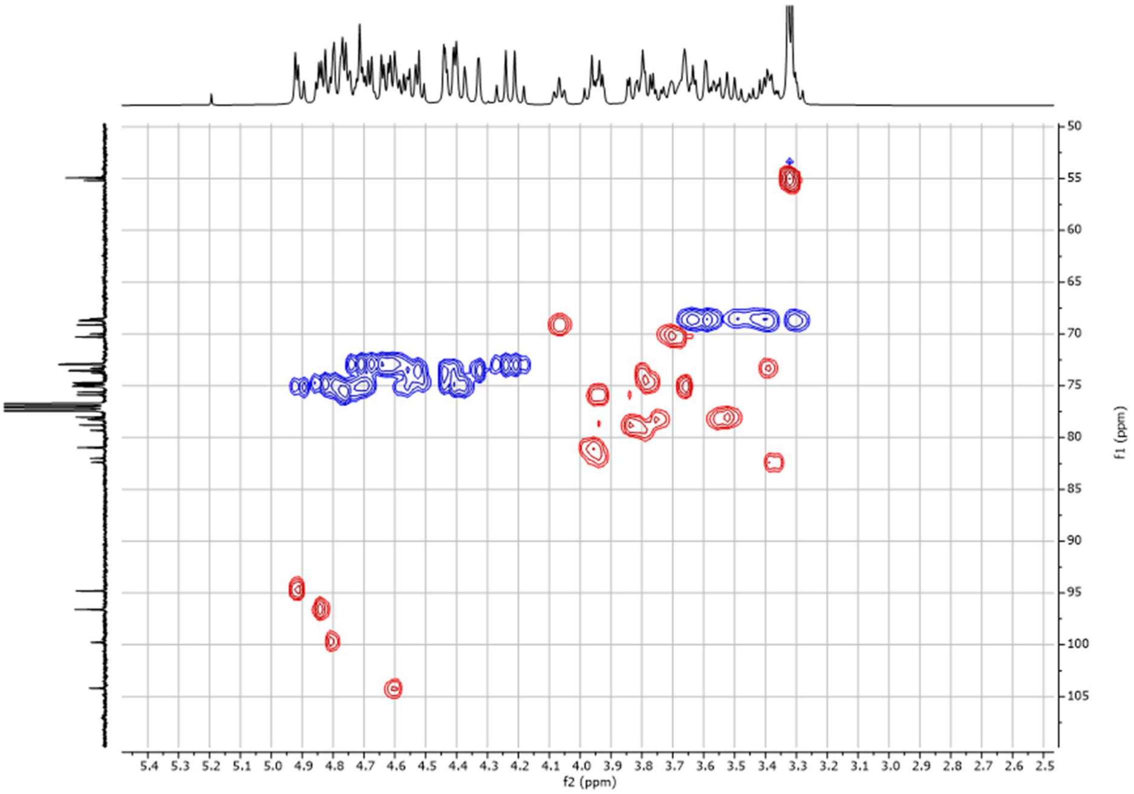


**Figure S4.** HSQC NMR Spectrum ($CDCl_3$, 400 MHz) of Compound **5**.

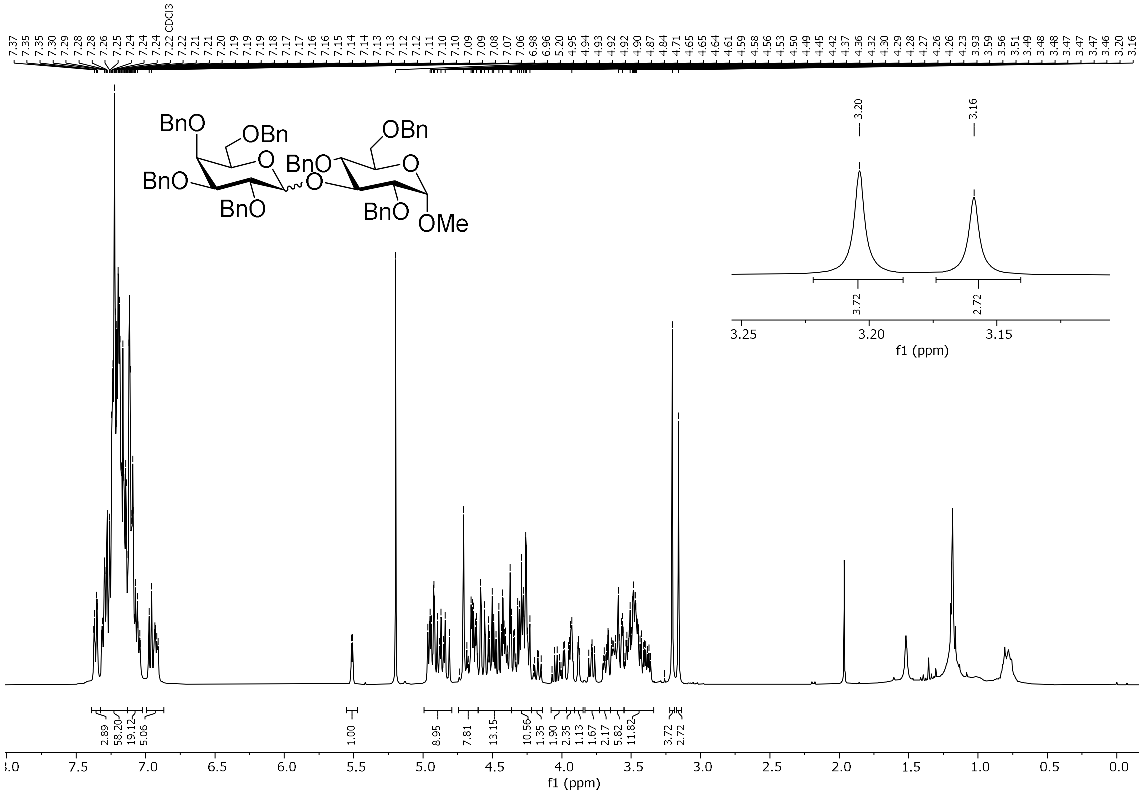

**Figure S5.** $^{1}H$ NMR Spectrum ($CDCl_3$, 400 MHz) of Compound **7.**

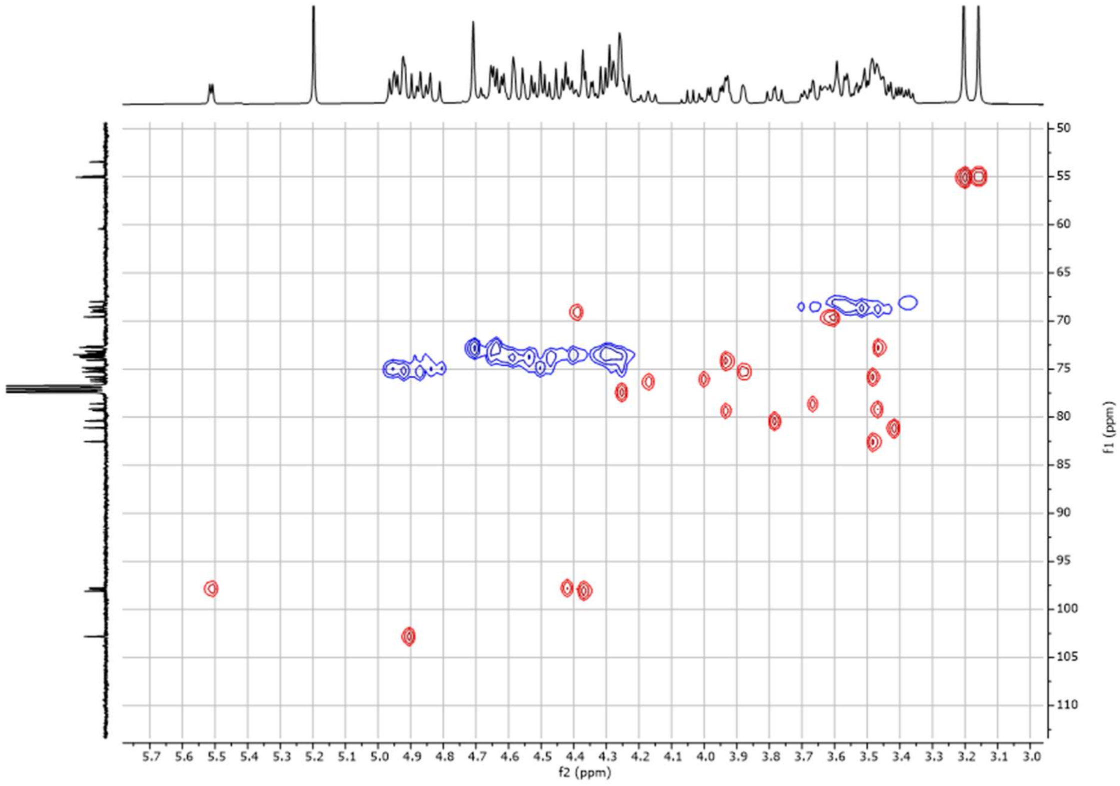

**Figure S6**. HSQC NMR Spectrum ($CDCl_3$, 400 MHz) of Compound **7**.

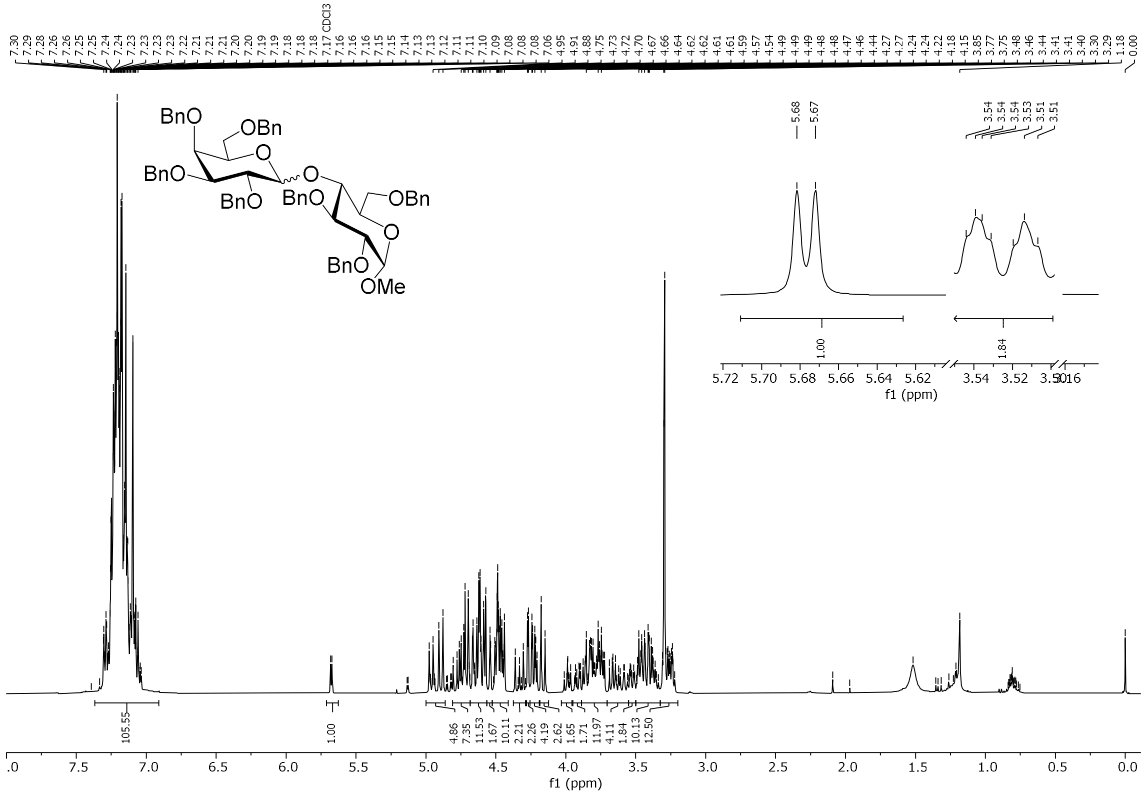

**Figure S7.** $^{1}$H NMR Spectrum ($CDCl_3$, 400 MHz) of Compound **9**.

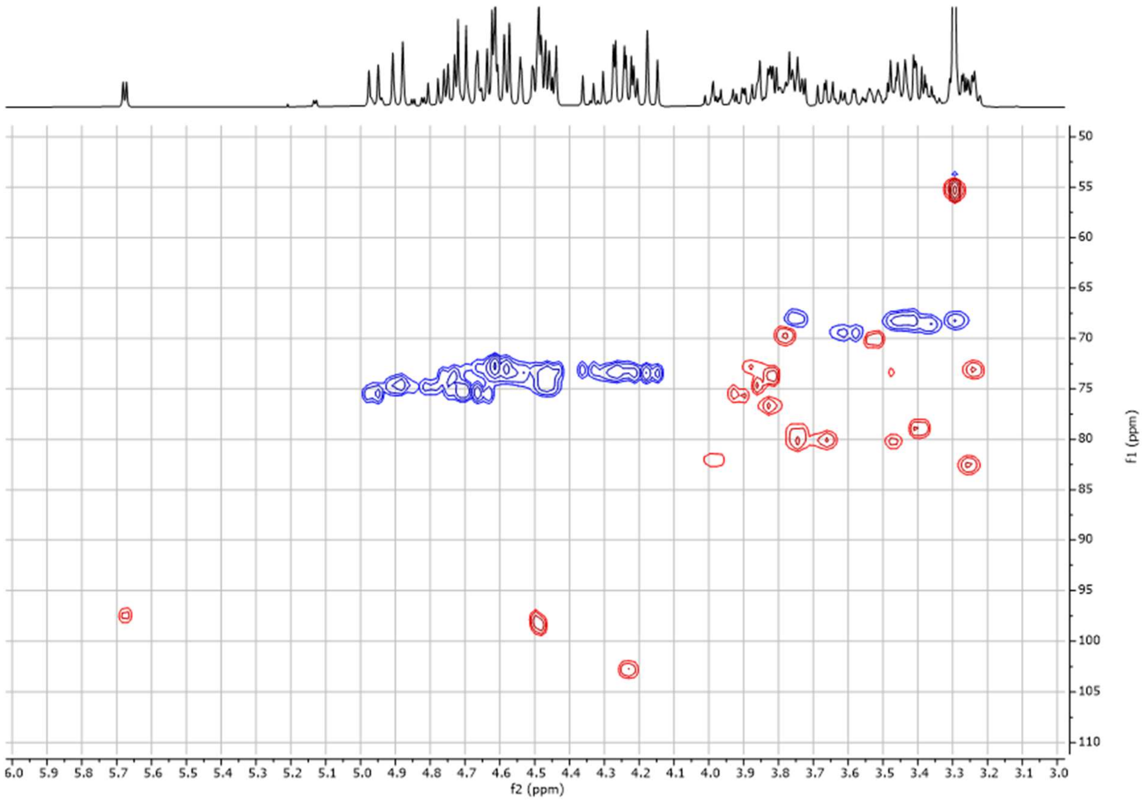

**Figure S8.** HSQC NMR Spectrum ($CDCl_3$, 400 MHz) of Compound **9**.

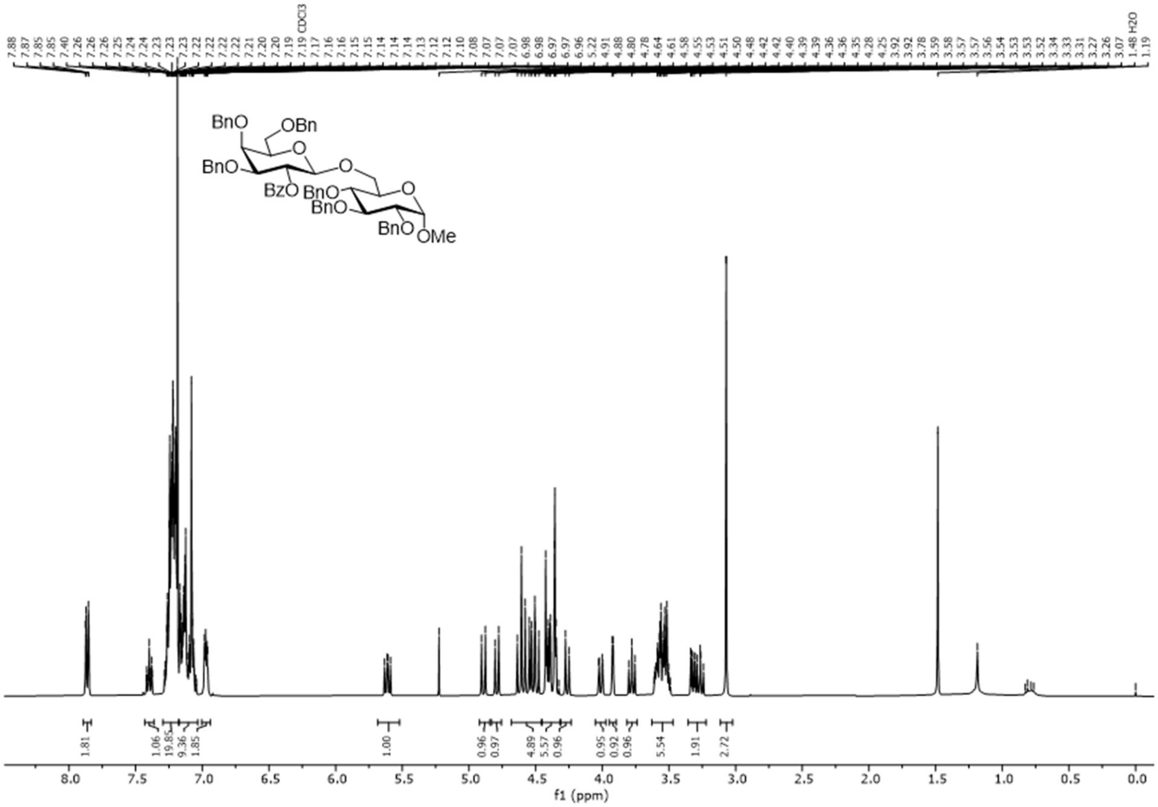


**Figure S9.** $^1$H NMR Spectrum ($CDCl_3$, 400 MHz) of Compound **11**.

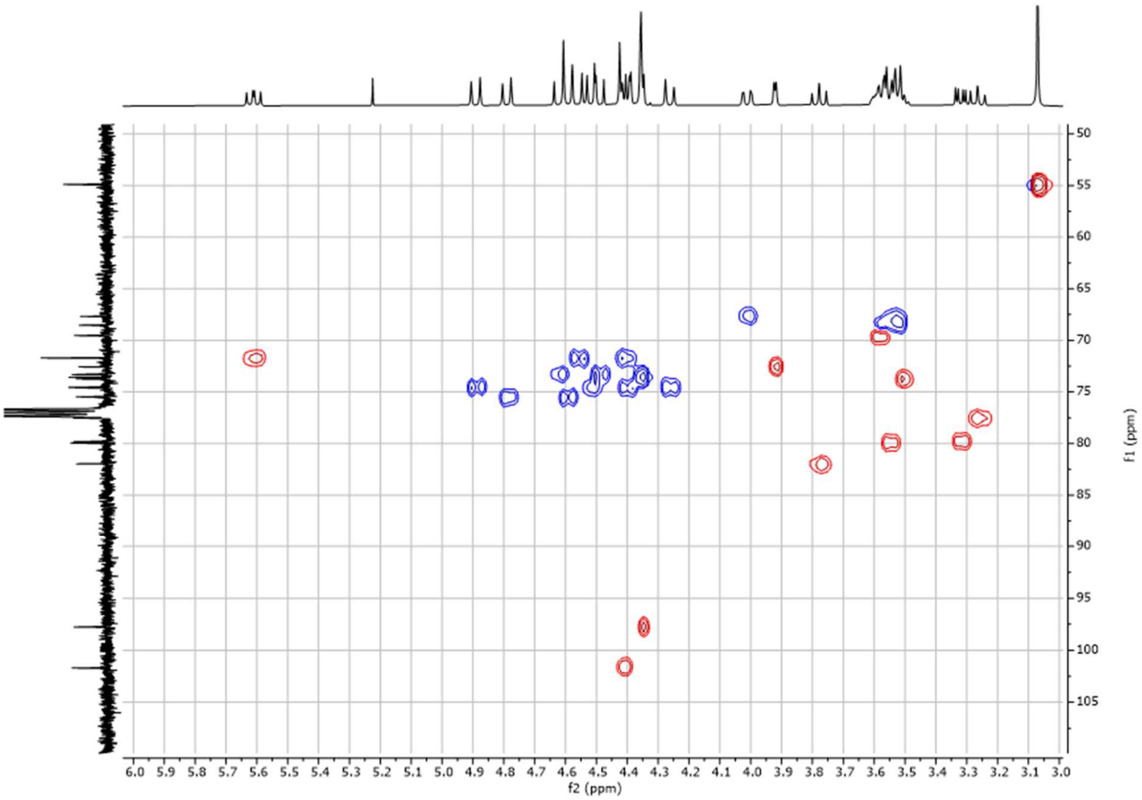


**Figure S10.** HSQC NMR Spectrum ($CDCl_3$, 400 MHz) of Compound **11**.

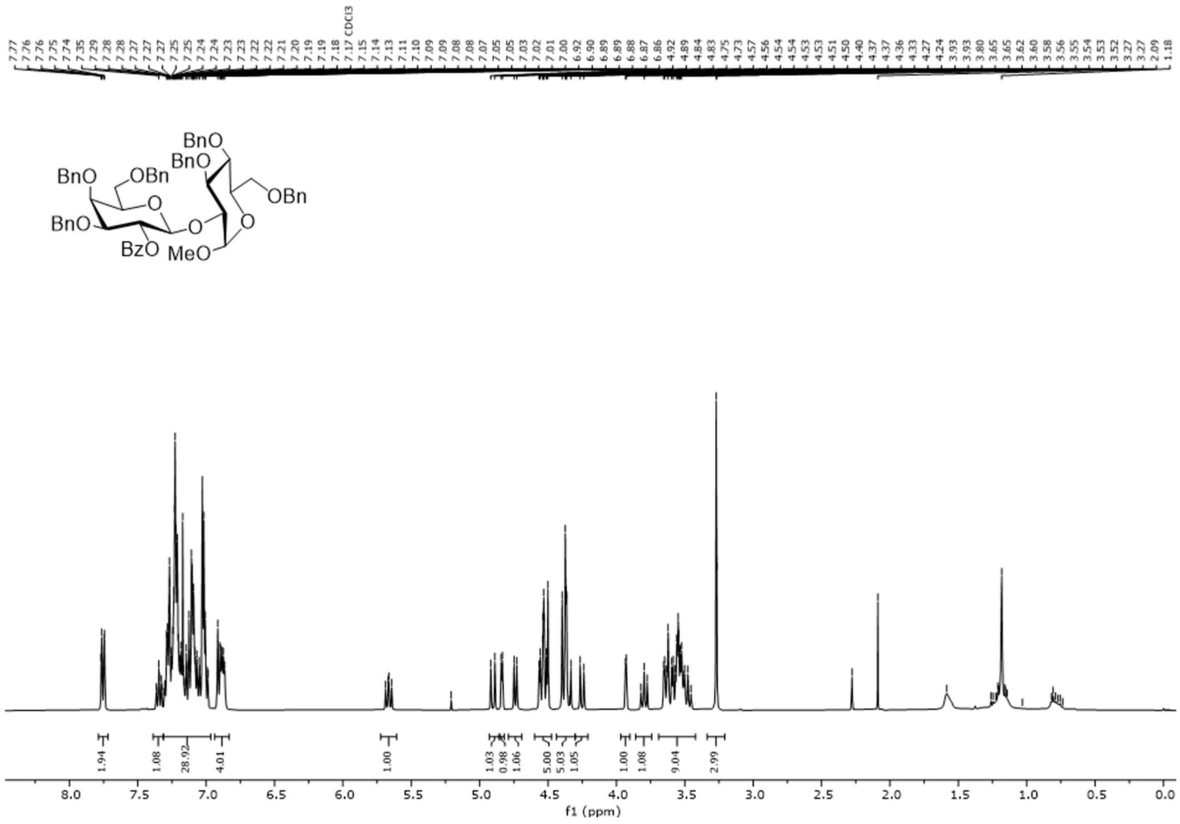


**Figure S11.** $^{1}H$ NMR Spectrum ($CDCl_3$, 400 MHz) of Compound **12**.

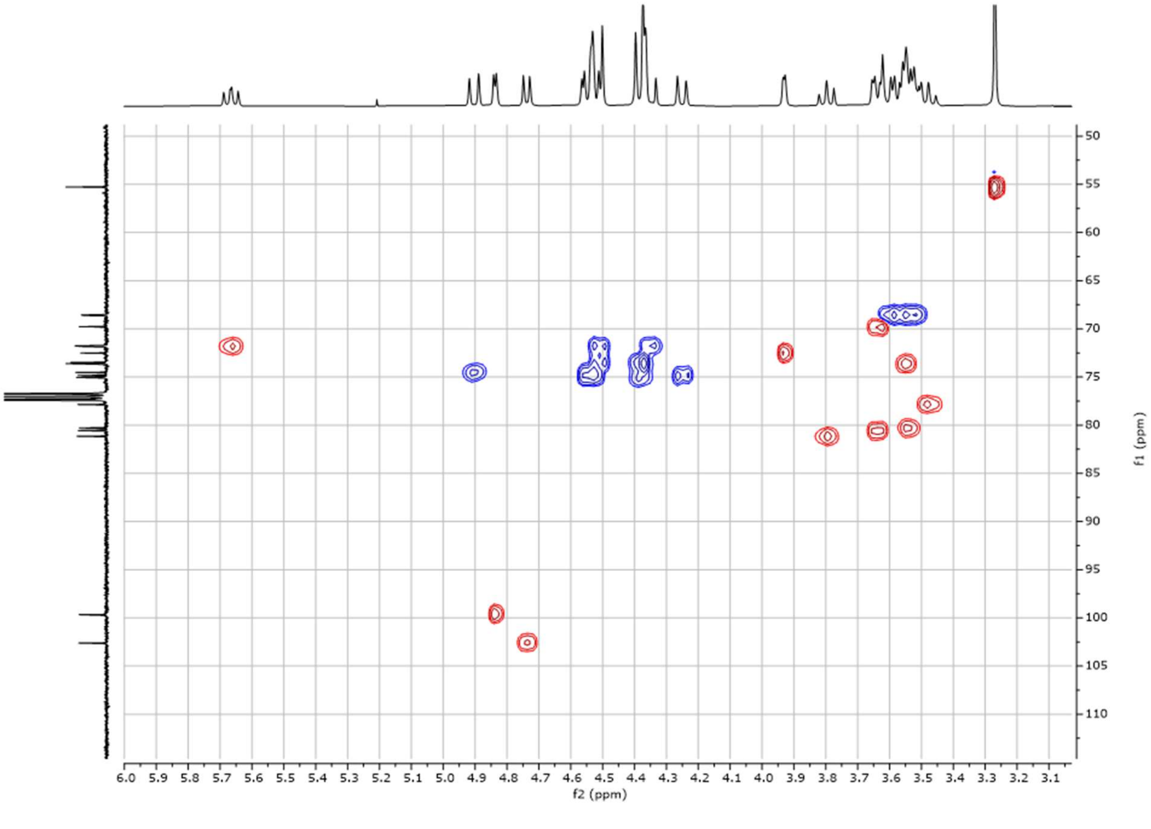


**Figure S12.** HSQC NMR Spectrum ($CDCl_3$, 400 MHz) of Compound **12**.

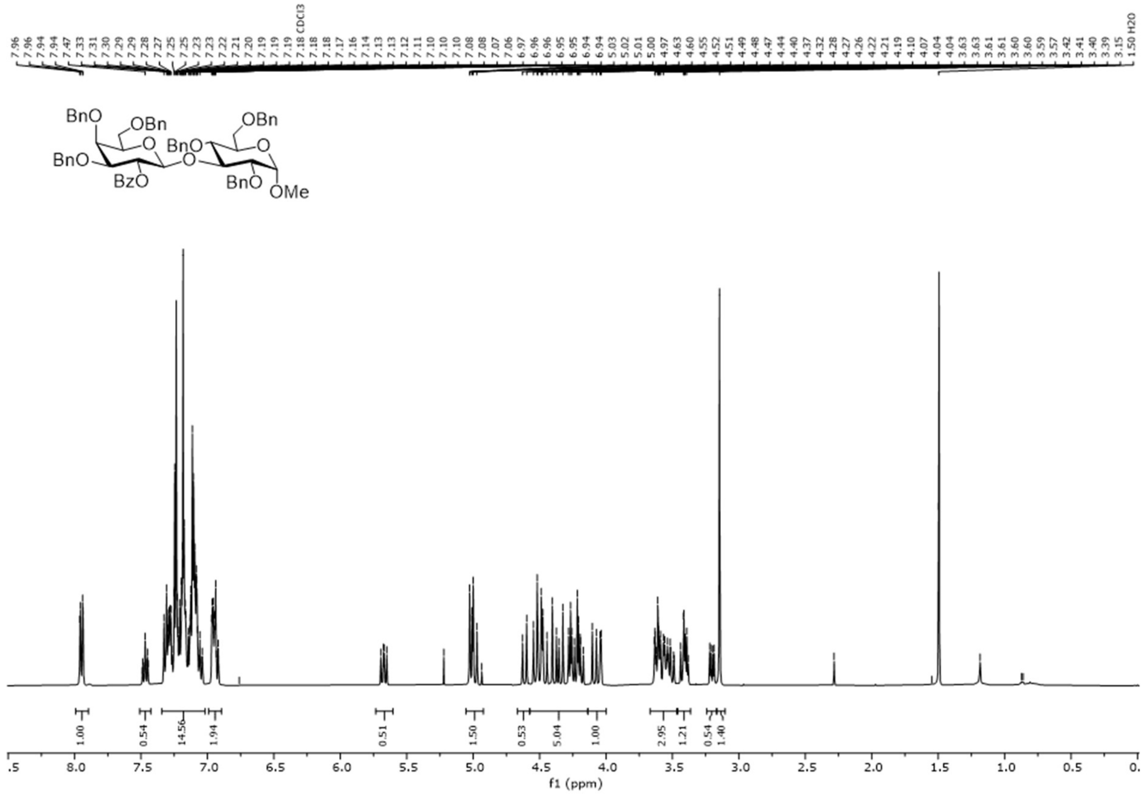


**Figure S13.** $^{1}H$ NMR Spectrum ($CDCl_3$, 400 MHz) of Compound **13**.

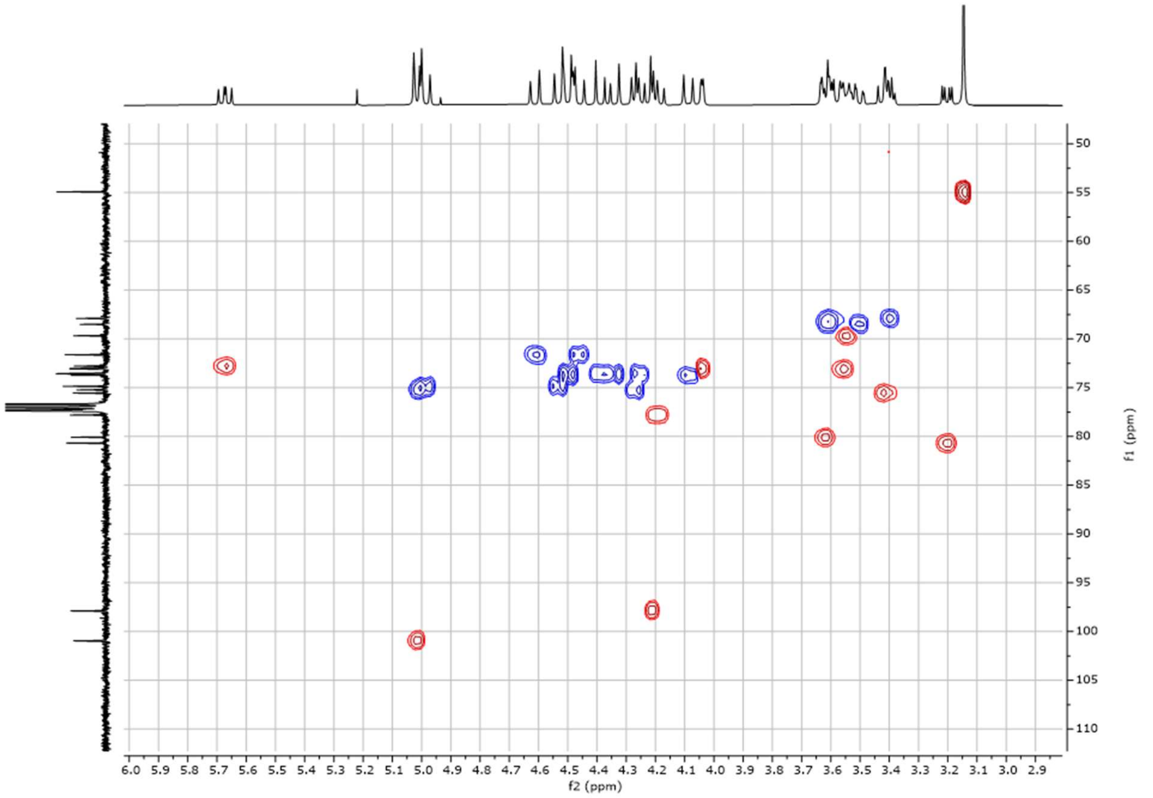


**Figure S14.** HSQC NMR Spectrum ($CDCl_3$, 400 MHz) of Compound **13**.

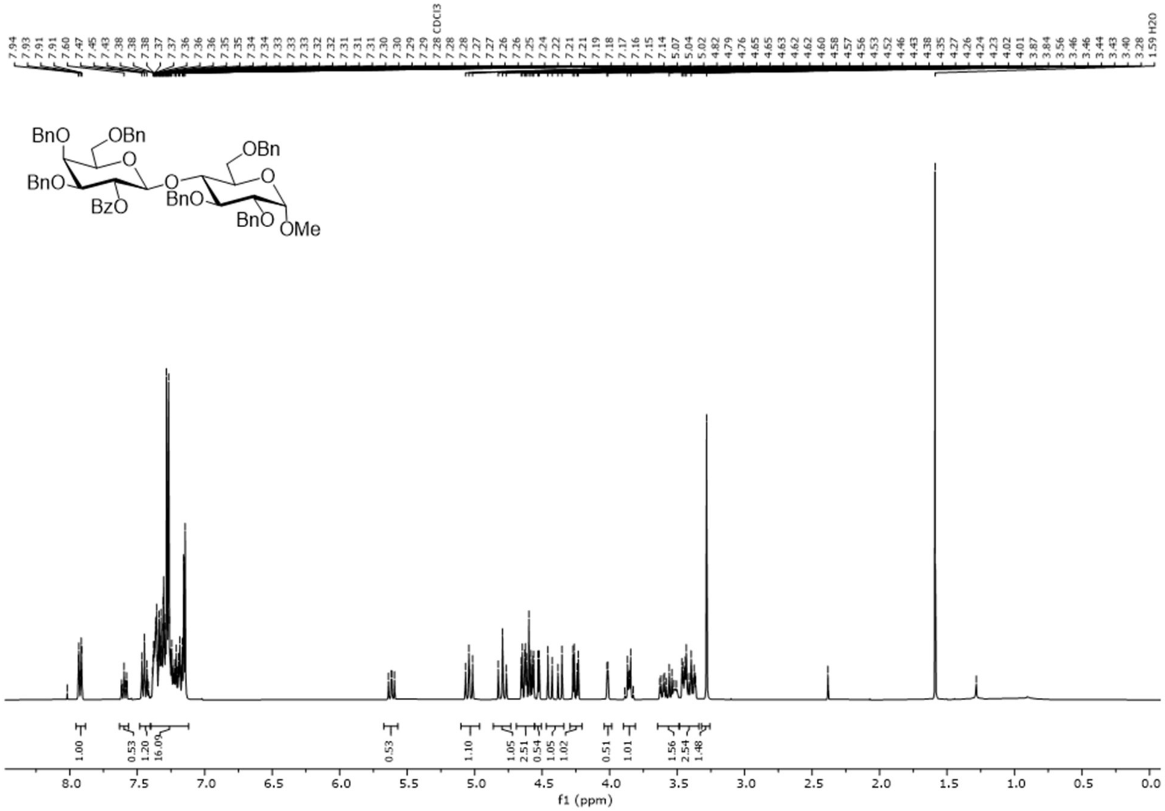


**Figure S15.** $^{1}$H NMR Spectrum of ($CDCl_3$, 400 MHz) Compound **14**.

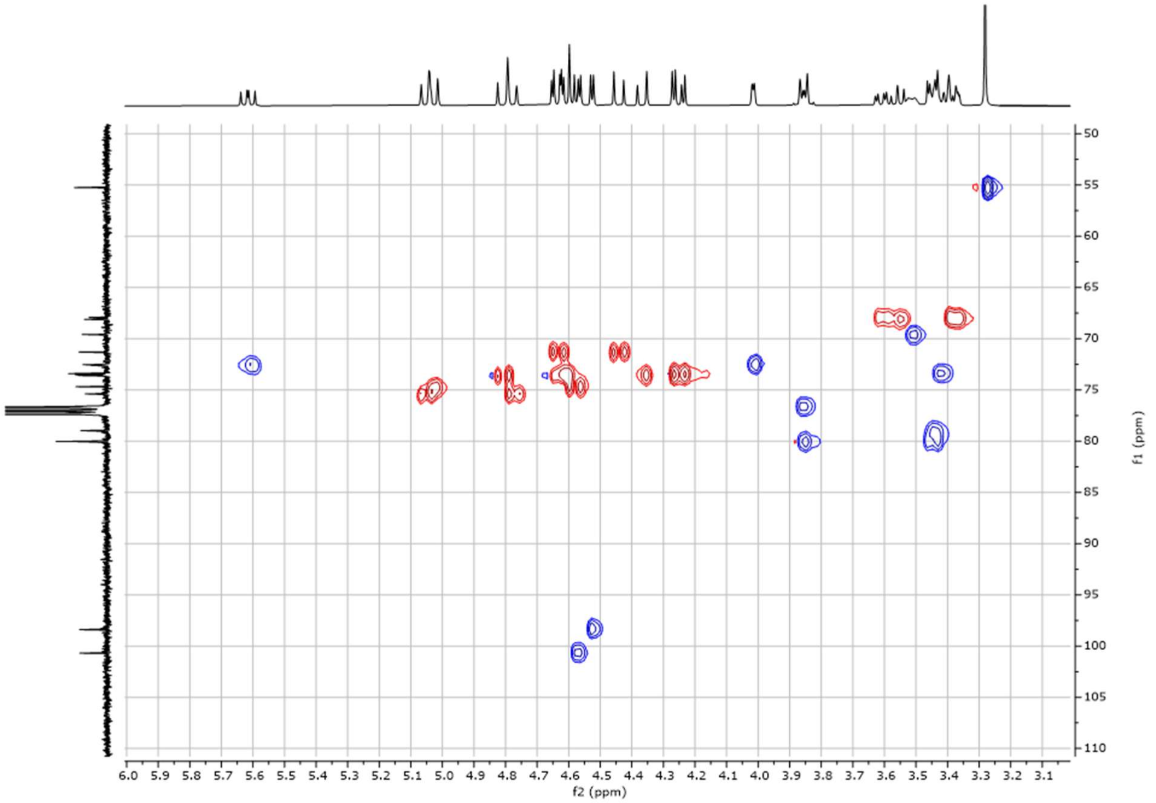


**Figure S16.** HSQC NMR Spectrum ($CDCl_3$, 400 MHz) of Compound **14**.

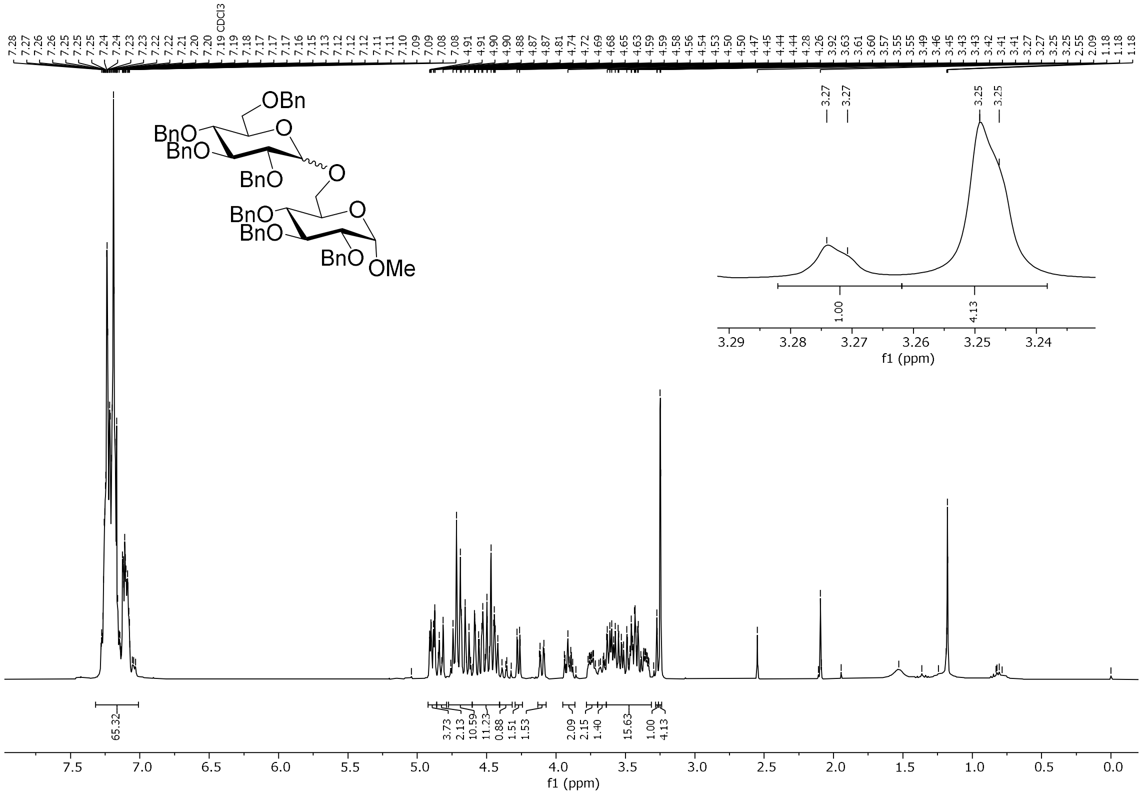

**Figure S17.** $^{1}$H NMR Spectrum ($CDCl_3$, 400 MHz) of Compound **16**.

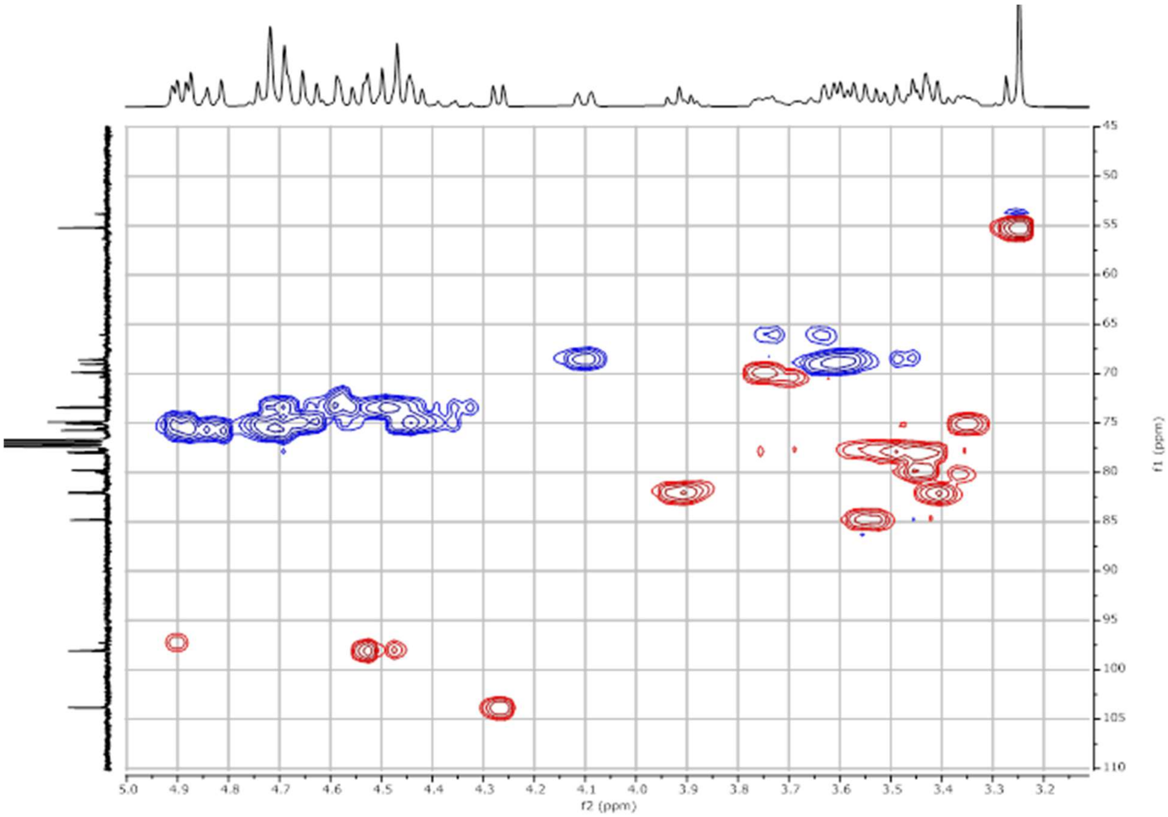

**Figure S18.** HSQC NMR Spectrum ($CDCl_3$, 400 MHz) of Compound **16**.

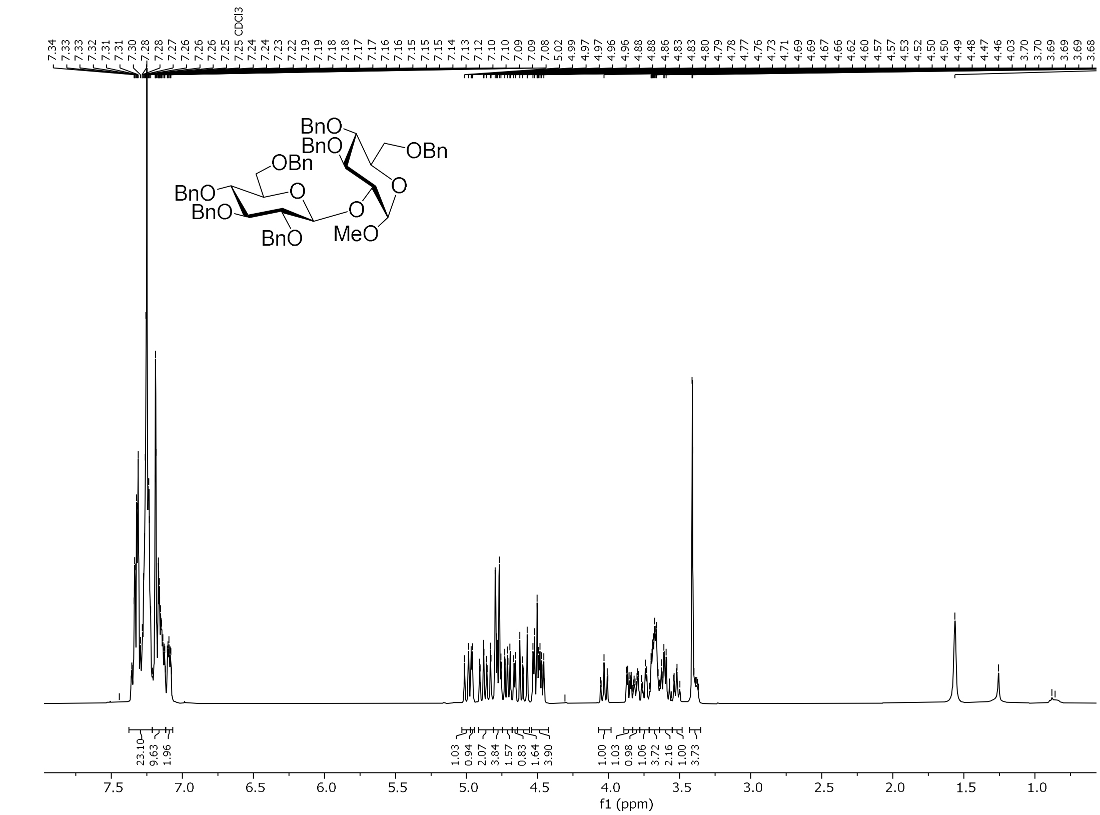


**Figure S19.** $^1$H NMR Spectrum ($CDCl_3$, 400 MHz) of Compound **17β**.

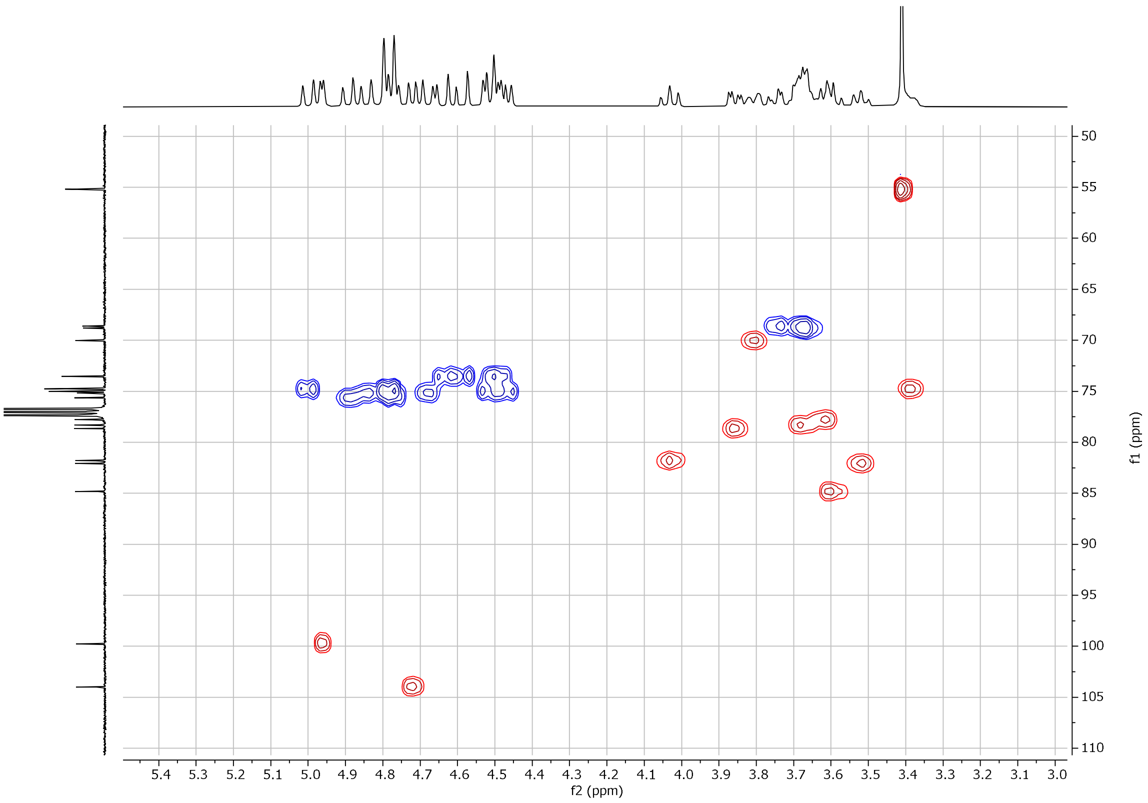


**Figure S20.** HSQC NMR Spectrum ($CDCl_3$, 400 MHz) of Compound **17β**.

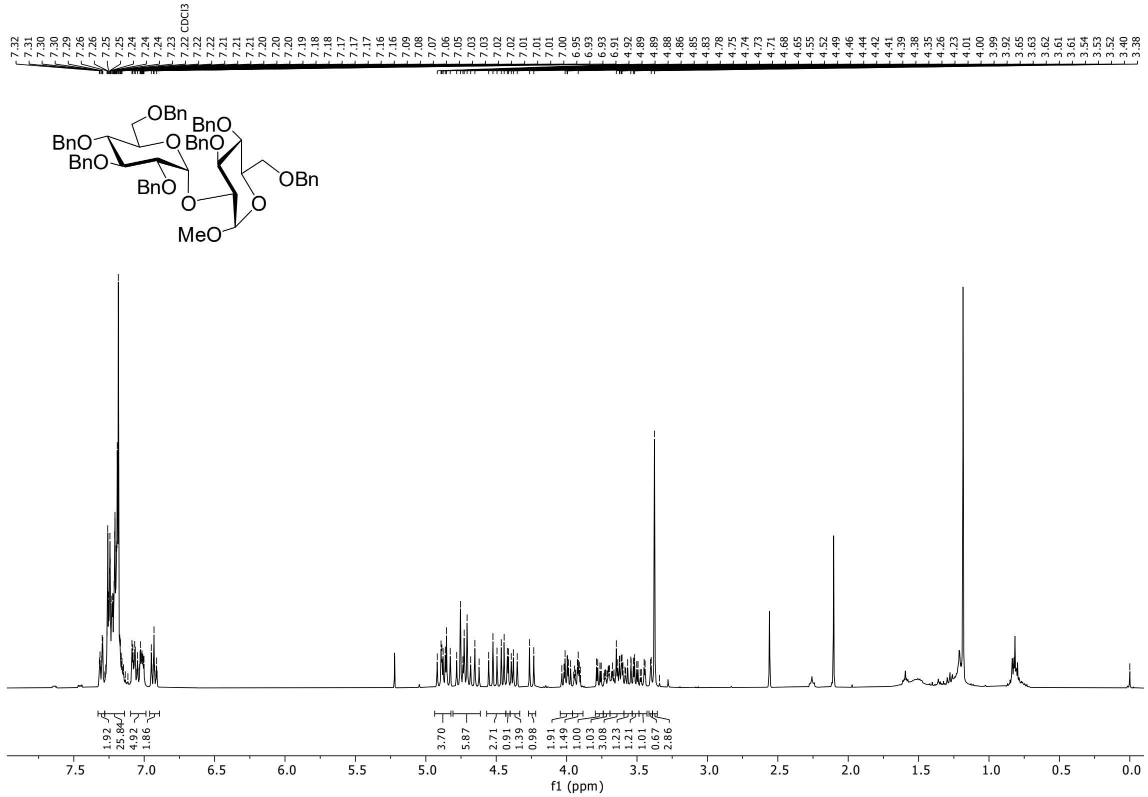


**Figure S21.** $^{1}$H NMR Spectrum ($CDCl_3$, 400 MHz) of Compound **17α**.

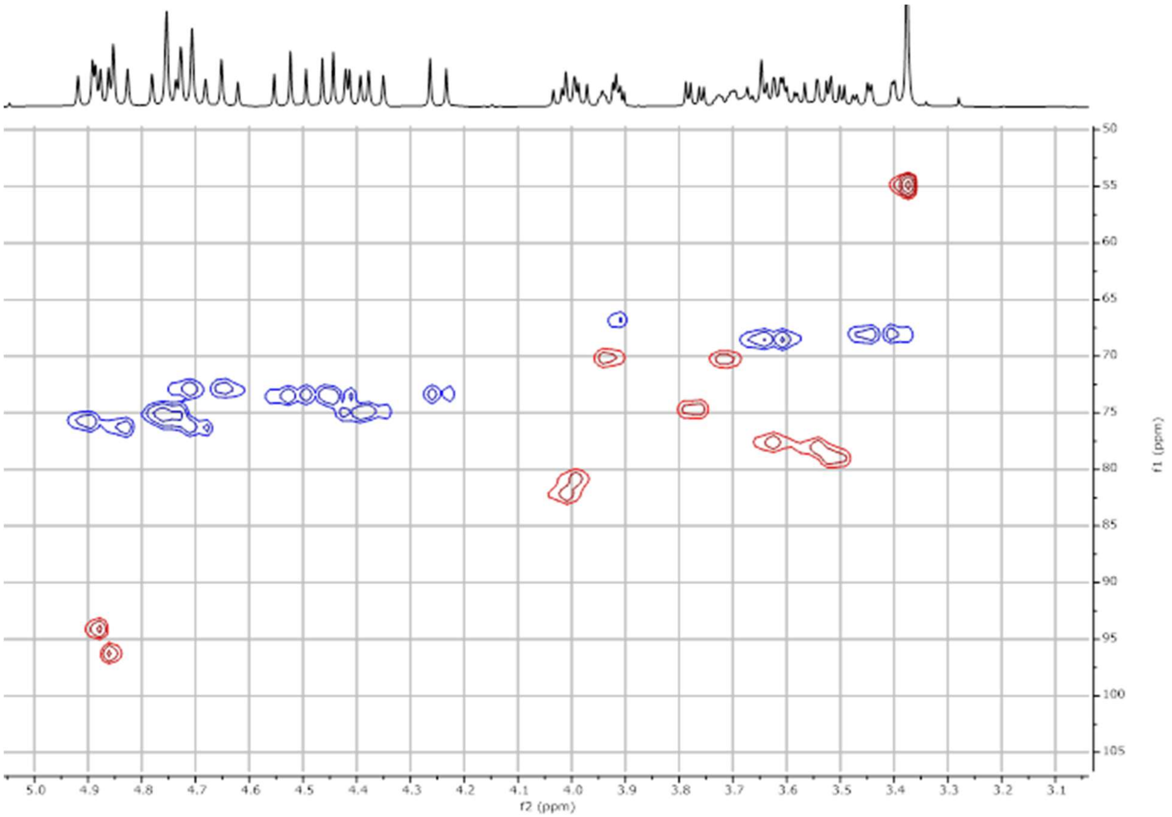


**Figure S22.** HSQC NMR Spectrum ($CDCl_3$, 400 MHz) of Compound **17α**.

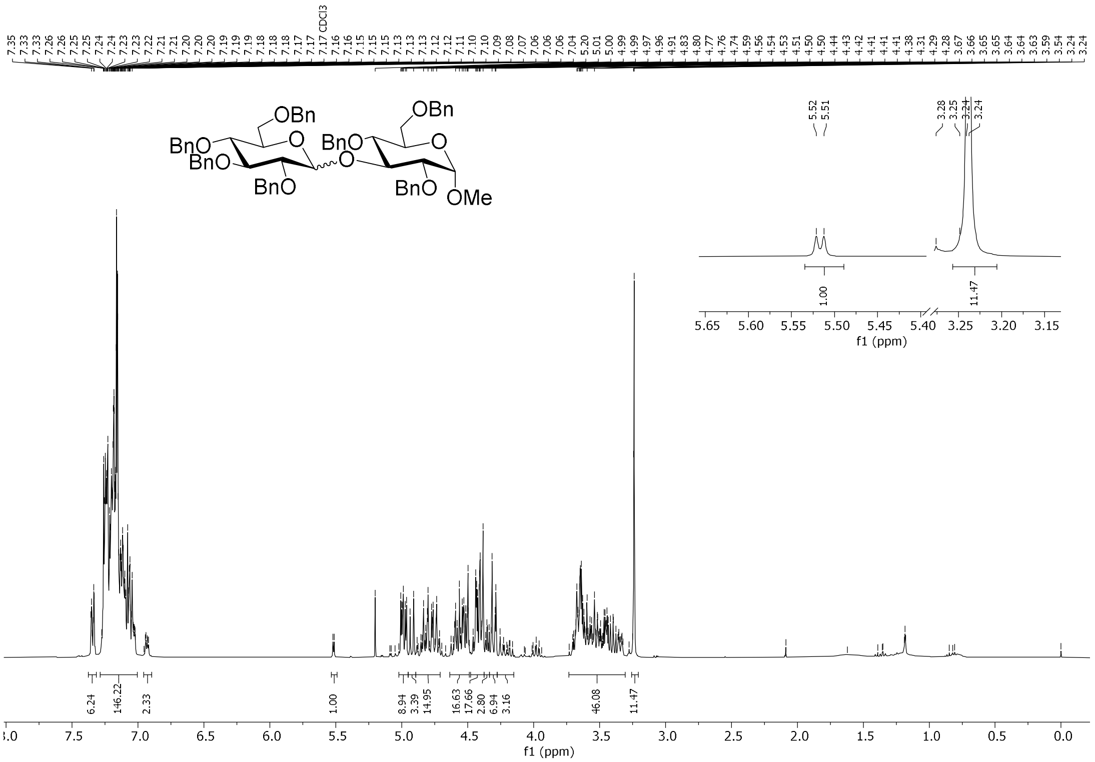


**Figure S23.** $^{1}H$ NMR Spectrum ($CDCl_3$, 400 MHz) of Compound **18**.

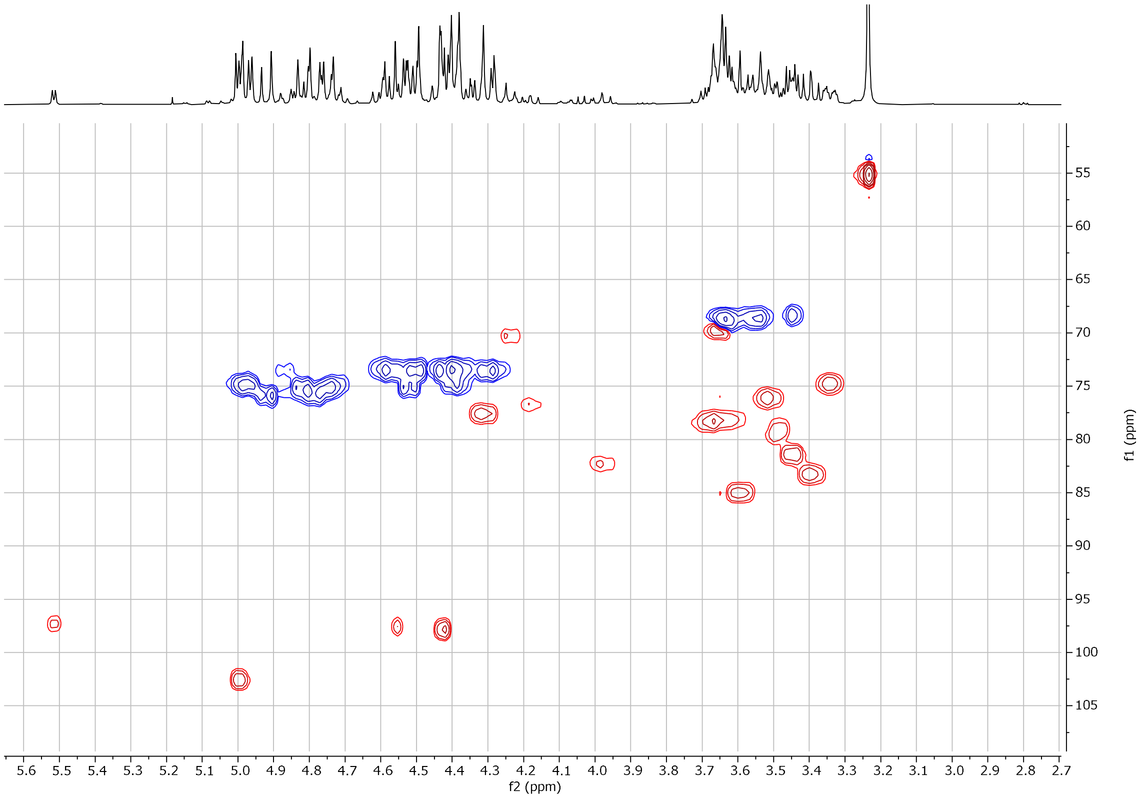


**Figure S24.** HSQC NMR Spectrum ($CDCl_3$, 400 MHz) of Compound **18**.

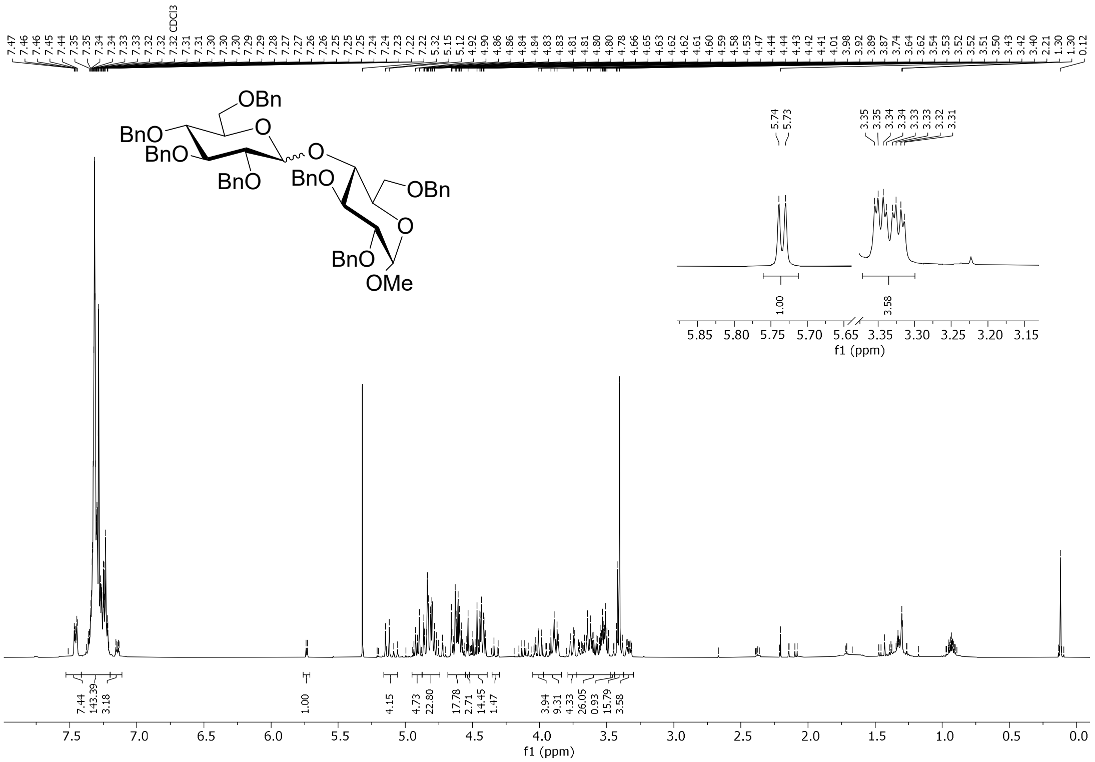

**Figure S25.** $^{1}H$ NMR Spectrum ($CDCl_3$, 400 MHz) of Compound **19**.

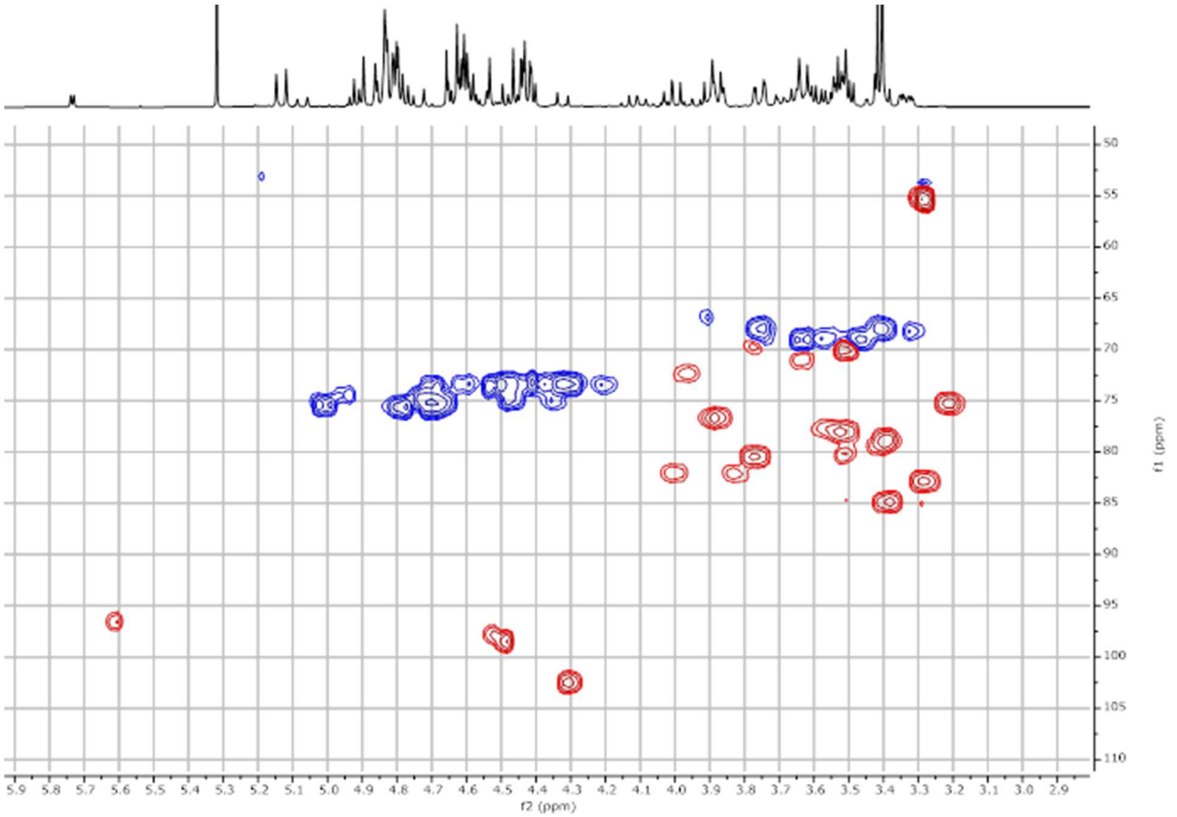

**Figure S26.** HSQC NMR Spectrum ($CDCl_3$, 400 MHz) of Compound **19** .

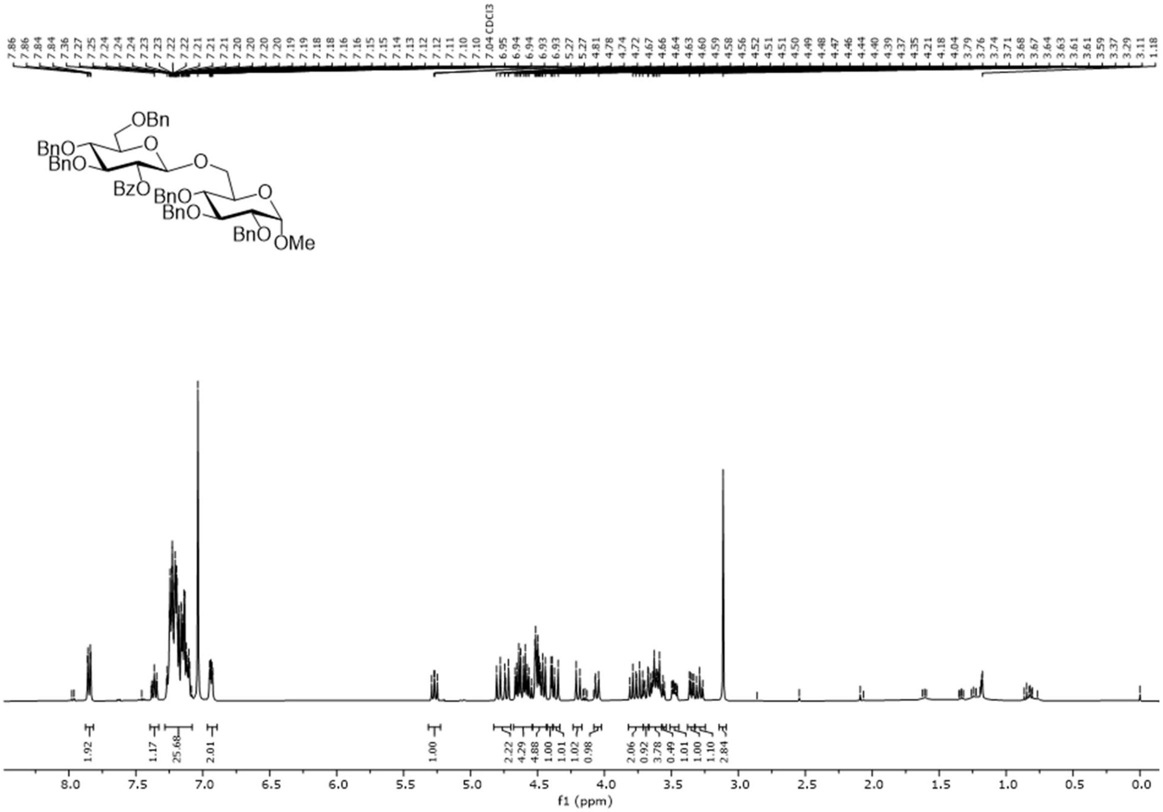


**Figure S27.** $^{1}$H NMR Spectrum ($CDCl_3$, 400 MHz) of Compound **21**.

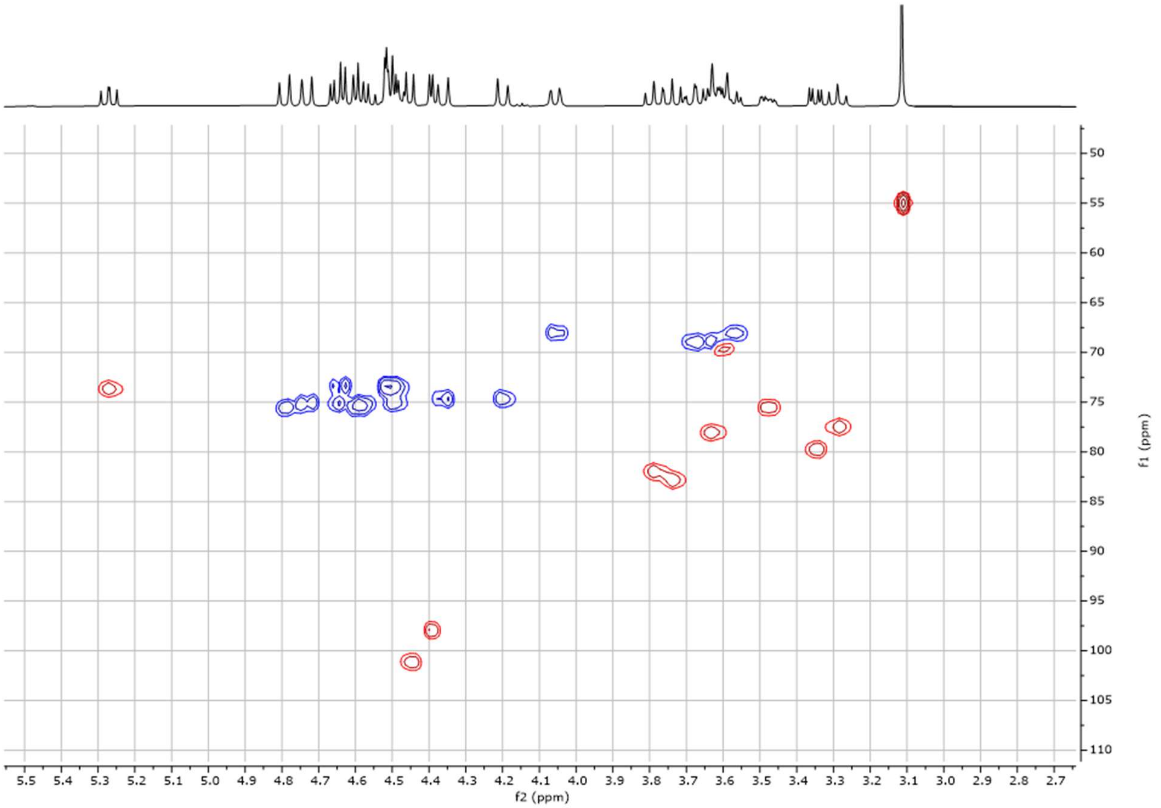


**Figure S28.** HSQC NMR Spectrum ($CDCl_3$, 400 MHz) of Compound **21**.

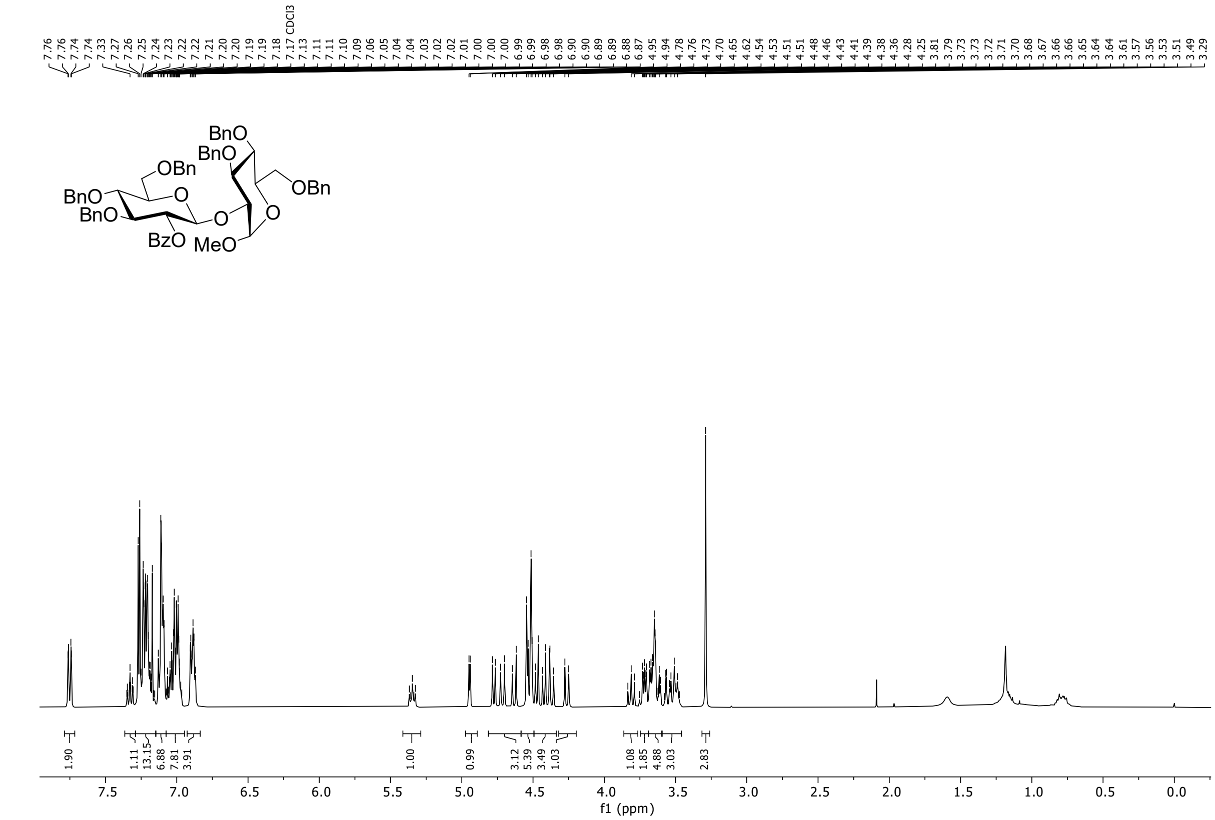


**Figure S29.** $^{1}$H NMR Spectrum ($CDCl_3$, 400 MHz) of Compound **22**.

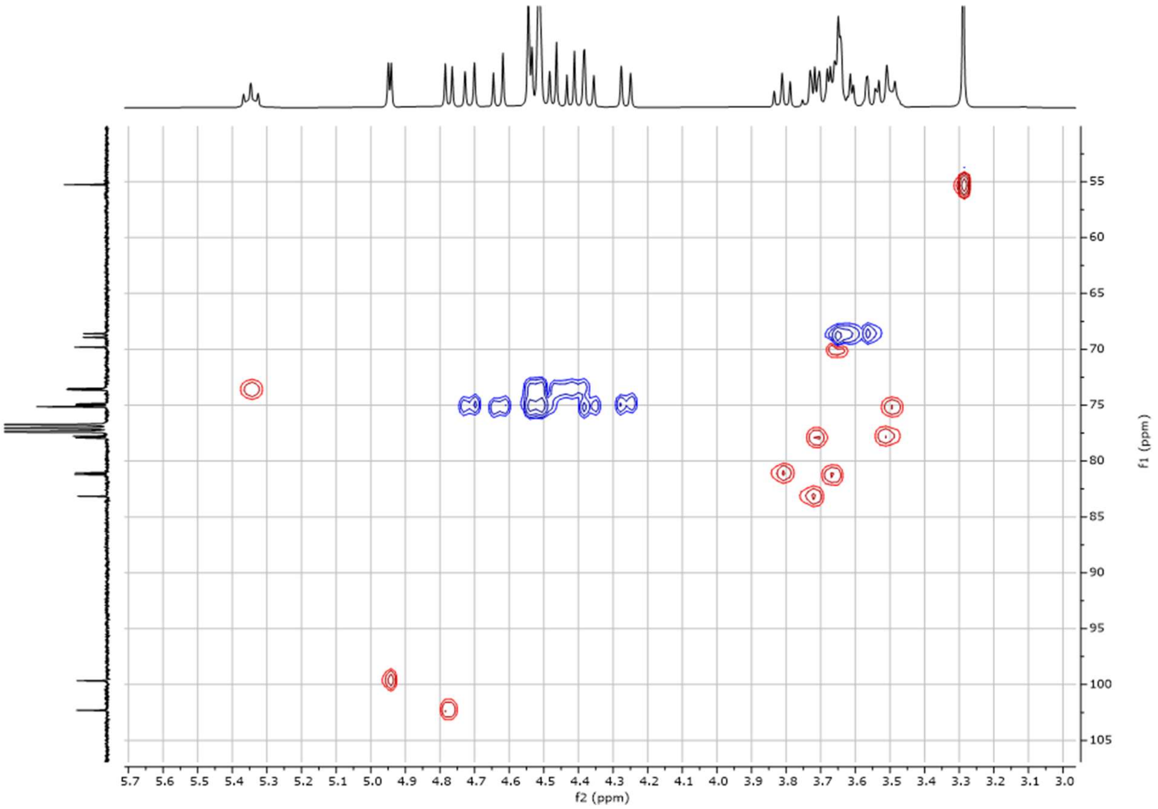


**Figure S30.** HSQC NMR Spectrum ($CDCl_3$, 400 MHz) of Compound **22**.

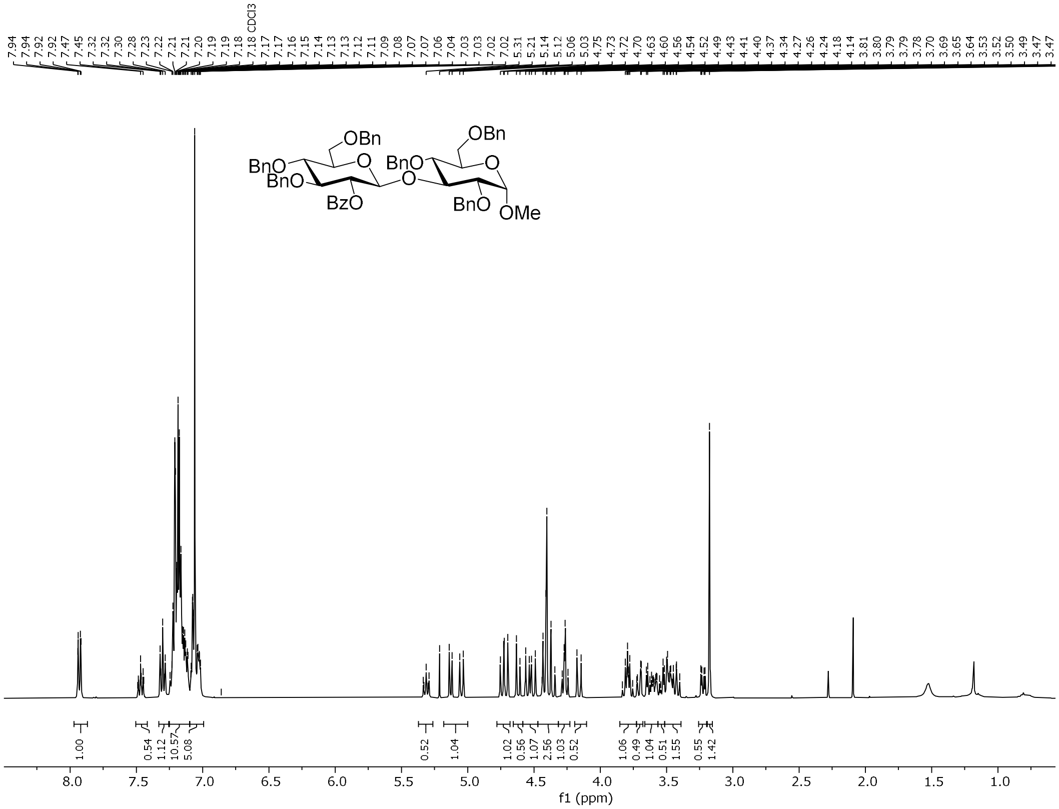


**Figure S31.** $^{1}H$ NMR Spectrum ($CDCl_3$, 400 MHz) of Compound **23**.

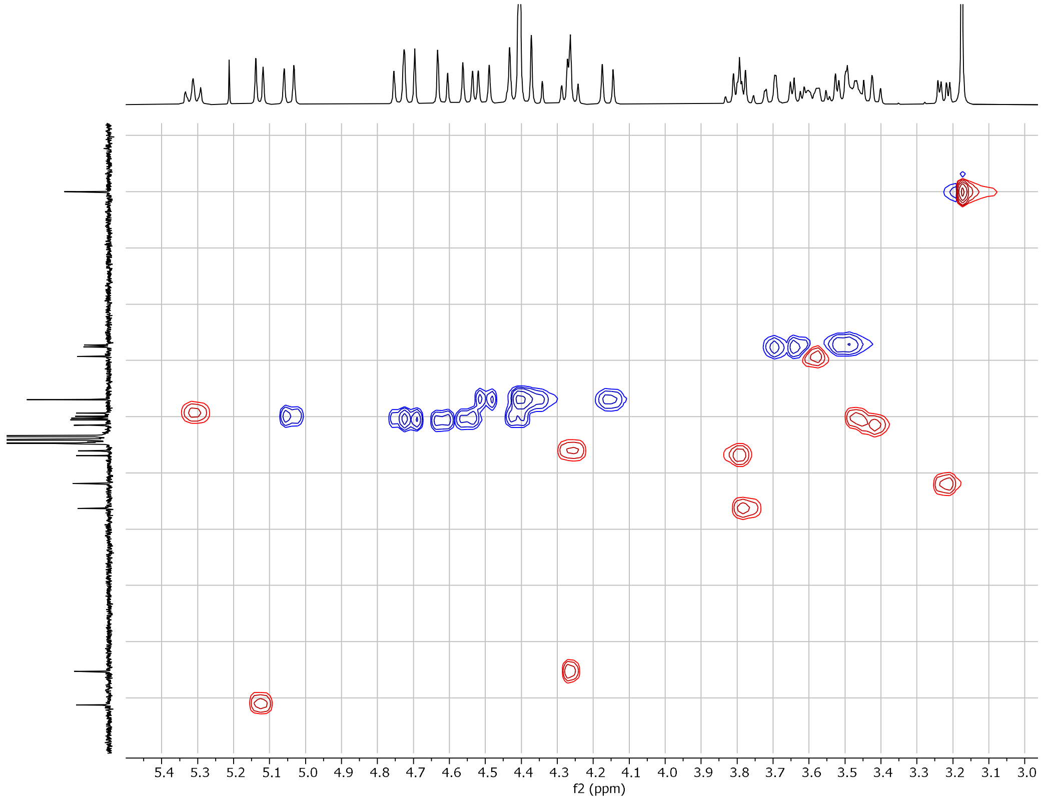


**Figure S32.** HSQC NMR Spectrum ($CDCl_3$, 400 MHz) of Compound **23**.

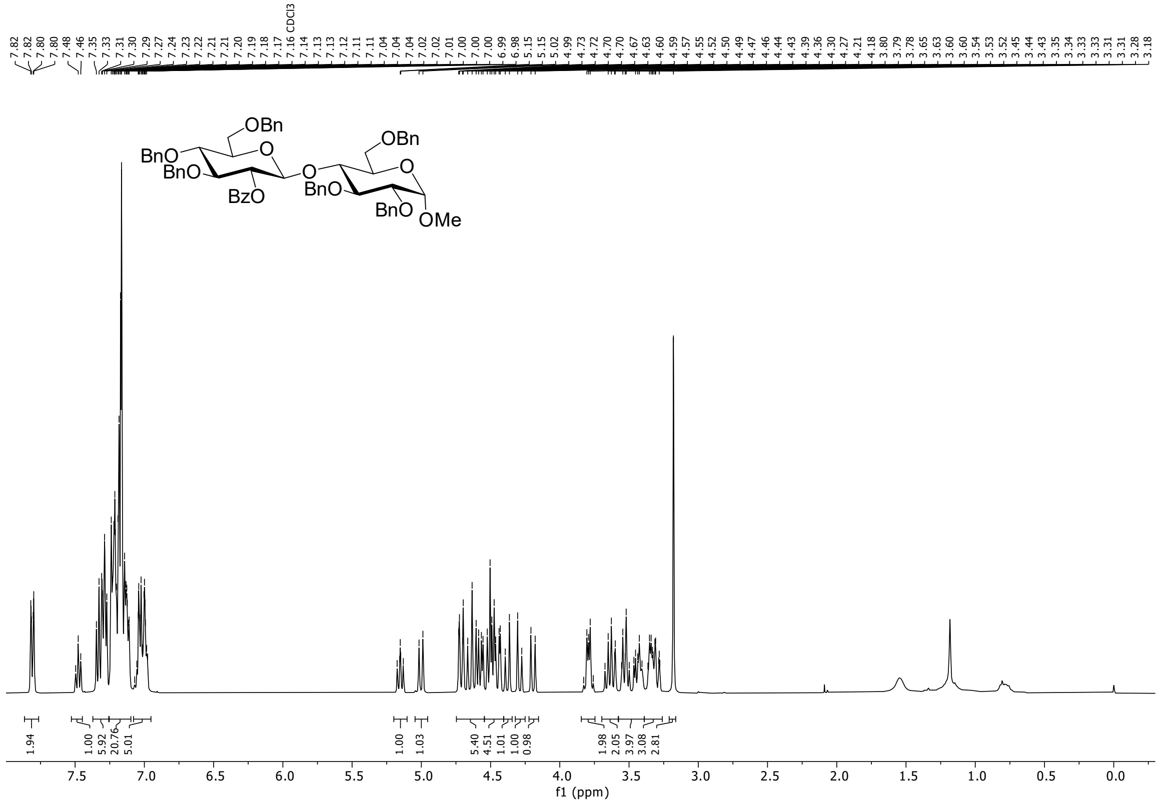


**Figure S33.** $^1$H NMR Spectrum ($CDCl_3$, 400 MHz) of Compound **24**.

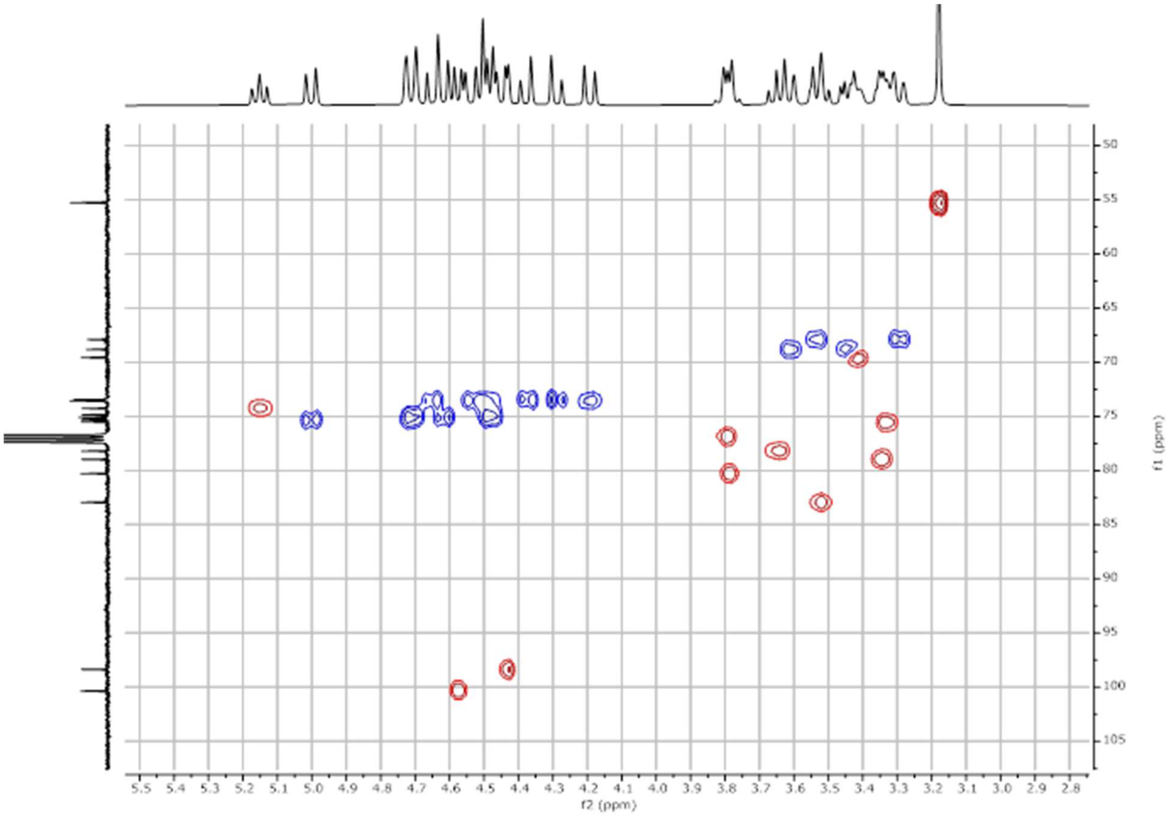


**Figure S34.** HSQC NMR Spectrum ($CDCl_3$, 400 MHz) of Compound **24**.

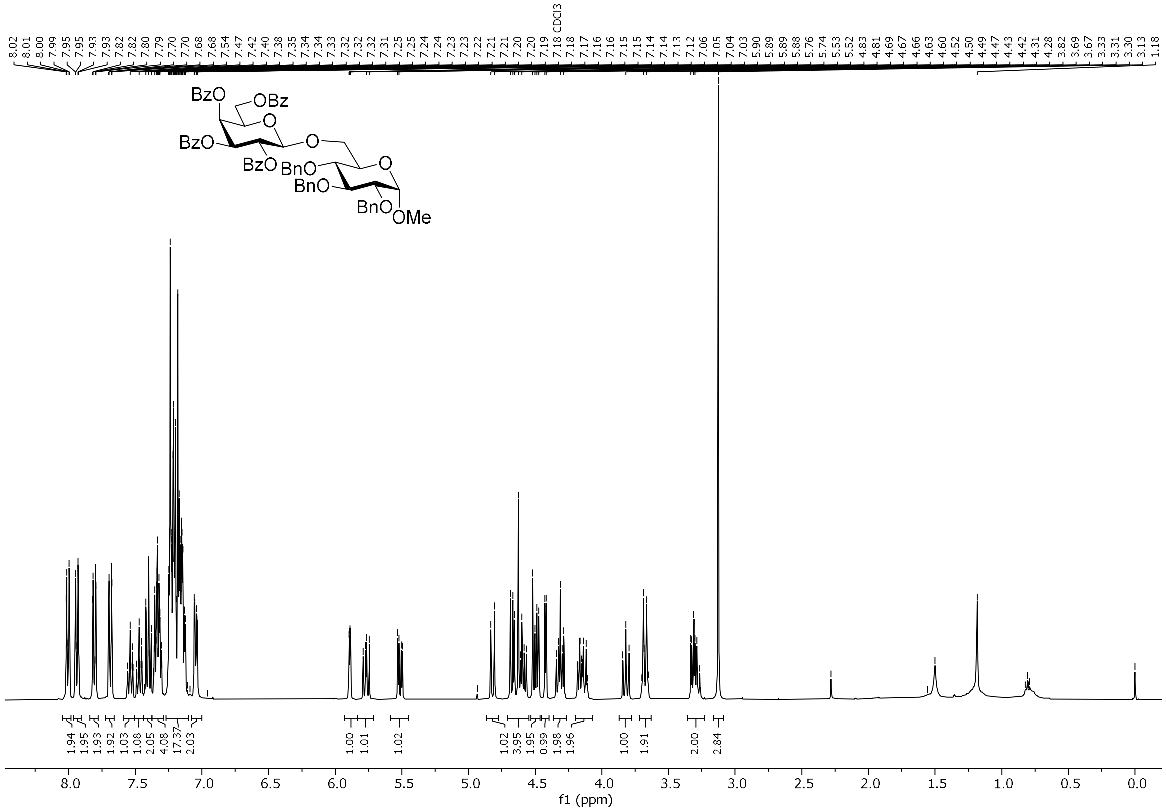

**Figure S35.** $^1H$ NMR Spectrum ($CDCl_3$, 400 MHz) of Compound **26**.

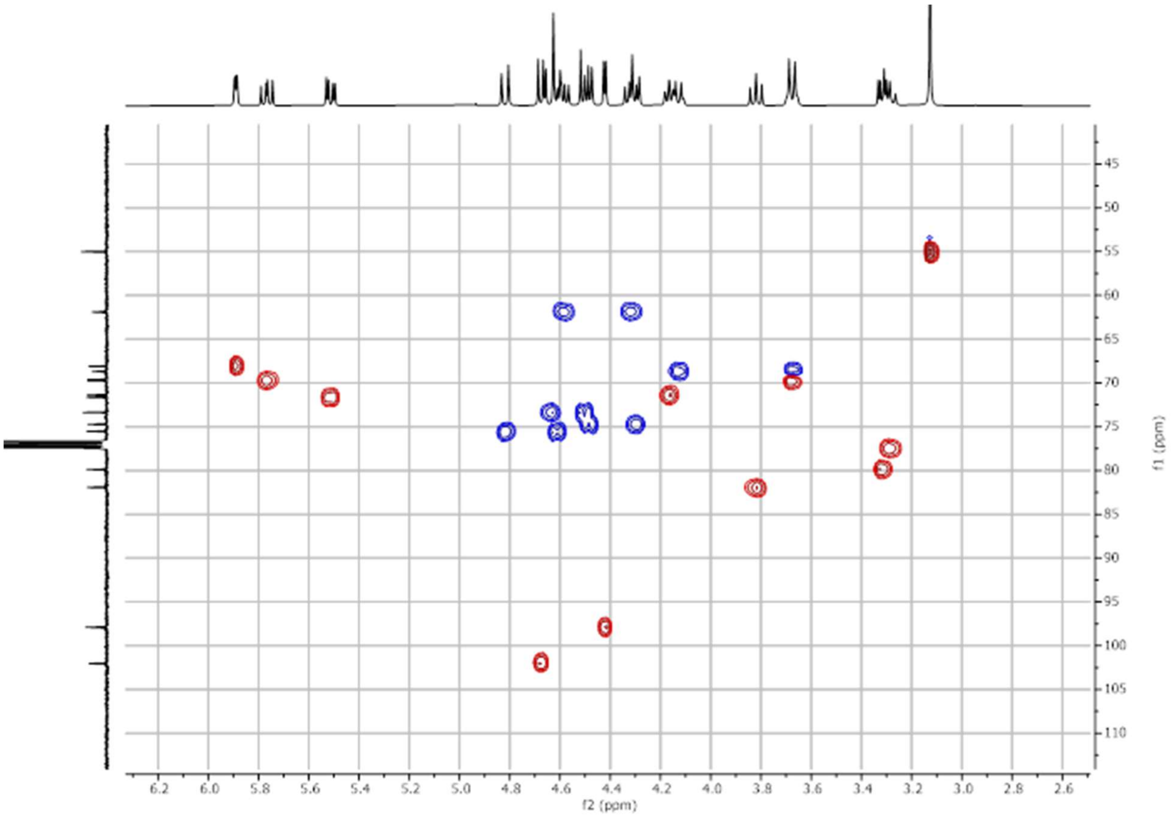

**Figure S36.** HSQC NMR Spectrum ($CDCl_3$, 400 MHz) of Compound **26**.
